# Treatment of *Pseudomonas aeruginosa* infectious biofilms: Challenges and strategies

**DOI:** 10.3389/fmicb.2022.955286

**Published:** 2022-08-26

**Authors:** Rui Yin, Juanli Cheng, Jingyao Wang, Panxin Li, Jinshui Lin

**Affiliations:** Shaanxi Key Laboratory of Chinese Jujube, College of Life Sciences, Yan'an University, Yan'an, Shaanxi Province, China

**Keywords:** *Pseudomonas aeruginosa*, antibiotic resistance, biofilm, anti-biofilm molecules, alternative therapeutics

## Abstract

*Pseudomonas aeruginosa*, a Gram-negative bacterium, is one of the major pathogens implicated in human opportunistic infection and a common cause of clinically persistent infections such as cystic fibrosis, urinary tract infections, and burn infections. The main reason for the persistence of *P. aeruginosa* infections is due to the ability of *P. aeruginosa* to secrete extracellular polymeric substances such as exopolysaccharides, matrix proteins, and extracellular DNA during invasion. These substances adhere to and wrap around bacterial cells to form a biofilm. Biofilm formation leads to multiple antibiotic resistance in *P. aeruginosa*, posing a significant challenge to conventional single antibiotic therapeutic approaches. It has therefore become particularly important to develop anti-biofilm drugs. In recent years, a number of new alternative drugs have been developed to treat *P. aeruginosa* infectious biofilms, including antimicrobial peptides, quorum-sensing inhibitors, bacteriophage therapy, and antimicrobial photodynamic therapy. This article briefly introduces the process and regulation of *P. aeruginosa* biofilm formation and reviews several developed anti-biofilm treatment technologies to provide new directions for the treatment of *P. aeruginosa* biofilm infection.

## Introduction

Most microorganisms have different survival mechanisms when facing stress conditions, such as proteolytic systems and growth regulation (Kumar et al., 2017). Most bacteria live in biofilms, which provide a protective niche for the survival of microorganisms (Kumar et al., 2017; Flemming and Wuertz, 2019). Bacterial biofilms are defined as structured microbial communities encapsulated in a self-synthesizing extracellular polymeric substance (EPS) and attached to a tissue or surface (Costerton et al., 1999) that include exopolysaccharides, matrix proteins, and extracellular DNA (eDNA; Wang et al., 2020). Clinically, the long-term colonization of bacteria in humans causes chronic infections, mainly because the bacteria in biofilms are resistant to host immune responses and antibiotic therapy (Dufour et al., 2010). Research has shown that 65–80% of pathogenic infections in hospitals are associated with biofilms (Kumar et al., 2017; Shrestha et al., 2022). Although numerous antimicrobial agents are available for clinical use, these agents only inhibit infection symptoms and are unable to eradicate bacteria embedded in biofilms (Li et al., 2020).

*Pseudomonas aeruginosa* is a Gram-negative opportunistic pathogenic bacterium that is widely found in nature. It has been established that *P. aeruginosa* is involved in a diverse array of infections, both community- and hospital-acquired, including pneumonia, cystic fibrosis, urinary tract infections, and burn infections (Al-Dahmoshi et al., 2021). Antimicrobial agents approved for clinical use may be ineffective in treating *P. aeruginosa* infections as this bacterium has the ability to form biofilms (Azam and Khan, 2019). The formation of biofilms enables *P. aeruginosa* to resist external adverse environments and enhance its colonization in the host. Biofilms can also act as diffusion barriers, restricting the entry of antibiotics into bacterial cells (Drenkard, 2003; Pang et al., 2019).

As *P. aeruginosa* is one of the major pathogens involved in opportunistic infections in humans, the clinical treatment and control of *P. aeruginosa* infections have become major challenges and have been the subject of extensive research (Azam and Khan, 2019; Das et al., 2020; Shao et al., 2020). The formation of biofilm effectively aids *P. aeruginosa* colonization, improving bacterial resistance to antimicrobial agents and countering the host immune system (Maurice et al., 2018; Elmanama et al., 2020; Tuon et al., 2022). Therefore, conventional single antibiotic therapy is limited in the treatment of biofilm infections, and an increasing number of studies have investigated the development of new antimicrobial drugs and anti-biofilm therapeutic programs to treat *P. aeruginosa* infection. This review will introduce the process of biofilm formation as well as biofilm regulation and anti-biofilm therapies in *P. aeruginosa*.

## Composition, formation, and regulation of *Pseudomonas aeruginosa* biofilm

The biofilm matrix components that have been identified from *P. aeruginosa* mainly include exopolysaccharides, eDNA, and matrix proteins, which play an important role in the structural maintenance and drug resistance of biofilms (Ghafoor et al., 2011). *P. aeruginosa* can synthesize at least three types of exopolysaccharides: alginate, Pel polysaccharide, and Psl polysaccharide (Mann and Wozniak, 2012). Alginate is an anionic polysaccharide of α-L-guluronic acid and β-D-mannuronic acid linked by β-1-4 glycosidic bonds (Moradali and Rehm, 2019). The overproduction of alginate is responsible for the development of excessive slimy or mucoid biofilms, while mucoid biofilms are more resistant to host immune system attack and antibiotic treatment than non-mucoid biofilms, resulting in persistent infections in the body (Moradali et al., 2017; Moradali and Rehm, 2019). The Psl polysaccharide consists of 15 co-transcribed genes (*pslA* to *pslO*) that encode proteins to synthesize Psl, enhance cell-surface and cell-to-cell adhesion in *P. aeruginosa*, and play an important role in the initiation and maintenance of biofilm structure (Ma et al., 2006). Pel is a positively charged exopolysaccharide composed of partially acetylated 1 → 4 glycosidic linkages of N-acetylgalactosamine and N-acetylglucosamine (Jennings et al., 2015), which is important for biofilm formation in air–liquid interfaces (Byrd et al., 2009). Pel and Psl are the major structural polysaccharides in non-mucoid and mucoid *P. aeruginosa* biofilms (Colvin et al., 2012; Ma et al., 2012). Cell lysis releases DNA into the environment, and this eDNA can be used as a supporting component of biofilms to provide nutrients to bacteria in biofilms during periods of nutrient deficiency (Finkel and Kolter, 2001). Aside from exopolysaccharides and eDNA, extracellular proteins are also considered to be important components of biofilm matrices, including appendages (mainly flagella and type IV fimbriae), cytoadhesions, and lectins. Studies have found that these components mainly play an auxiliary role as adhesion factors and structural support in the process of *P. aeruginosa* biofilm formation (Qi and Christopher, 2019).

**Figure 1:**
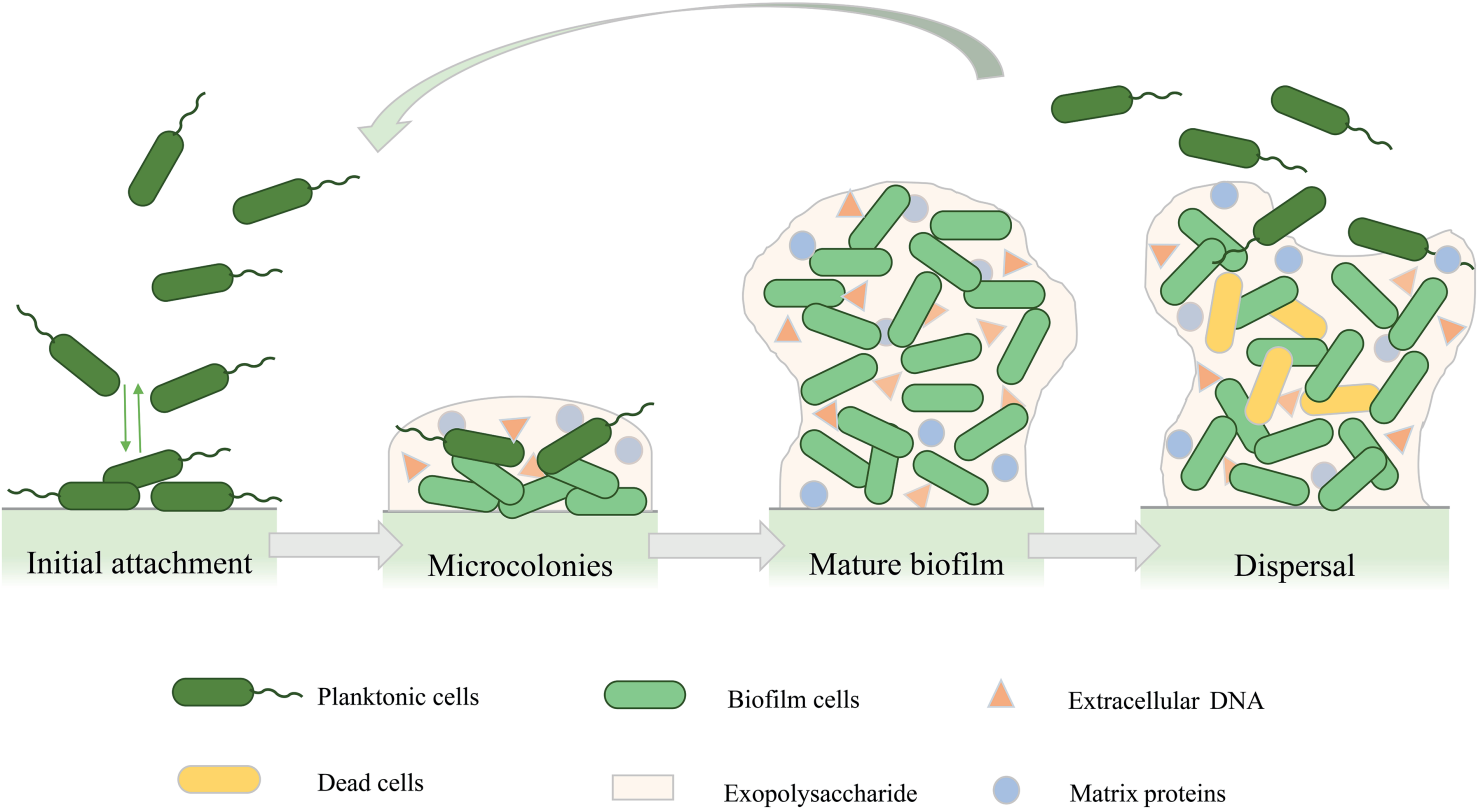
Cycle of *Pseudomonas aeruginosa* biofilm development. Biofilm formation includes four stages: initial attachment, microcolonies, mature biofilm, and biofilm dispersal.

The formation of the biofilm structure of *P. aeruginosa* is a continuous process that includes four main stages (Figure 1; Kostakioti et al., 2013; Saxena et al., 2019). The first stage is the initial attachment. At this stage, adhesion is reversible, and bacteria can attach to the surface or revert back to planktonic cells (Olivares et al., 2019). Many early studies on the initial attachment of bacteria have suggested that bacterial cells initiate adhesion through acid–base, hydrophobic, and electrostatic interactions (Donlan, 2002). In addition, the production of polysaccharides also contributes to cell-to-cell adhesion in *P. aeruginosa* (Ma et al., 2006). In the second stage of biofilm development, attached bacteria gradually reproduce into a more independent structure, and bacterial cells undergo a transition from reversible to irreversible attachment (Thi et al., 2020). In this stage, bacteria grow and form microcolonies and begin to synthesize EPSs, which acts as a blockade between biofilm cells and surroundings to provide protection from various stress conditions such as antimicrobial exposure or immune cell attack (Mitchell et al., 2016; Roy et al., 2018). As the secretion of EPSs continues, the cells forming microcolonies gradually mature and undergo various physiological changes (Lee and Yoon, 2017; Roy et al., 2018), forming three-dimensional mushroom-like structures consisting of small channels that transport nutrients, water, and waste, and contribute to the distribution of nutrients and signaling molecules as well as intercellular communication (Jamal et al., 2018; Thi et al., 2020). As the biofilm matures, the bacteria produce stronger resistance to environmental stress or antibiotics (Koo et al., 2017). The final stage of biofilm development is detachment. Different detachment mechanisms have been reported, such as erosion, sloughing, and dispersal (Kim and Lee, 2016). The detachment of biofilm marks the transition of biofilm cells to the planktonic mode of growth (Rumbaugh and Sauer, 2020), which leads cells to attach to the surface of other biomolecules or start a new cycle of biofilm development (Kim and Lee, 2016).

Matrix components of *P. aeruginosa* biofilms play different regulatory roles in different formation stages (Wei and Ma, 2013; Ma et al., 2022). In the early stage of biofilm formation, Psl polysaccharides can form a fibrous matrix that is spirally anchored to the surface of bacterial cells and wraps around bacteria, thus increasing the contact between bacteria and promoting the interaction between bacterial cells, resulting in the assembly and early adhesion of biofilms (Ma et al., 2006, 2009). eDNA is also considered to be an important factor in promoting the formation of *P. aeruginosa* biofilm and is involved in the initial attachment of bacterial cells as well as cell–cell interconnection (Allesen-Holm et al., 2006). During the later maturation stages of biofilms, Pel can serve a structural and protective role in the biofilm matrix of *P. aeruginosa* and enhance resistance to aminoglycoside antibiotics (Colvin et al., 2011). The production of numerous EPSs can promote biofilm growth through providing structural scaffolds and maintaining their biofilm function (Ghafoor et al., 2011). Furthermore, quorum sensing (QS), as a cell-density-dependent bacteria-cell signaling mechanism, plays a key role in the regulation of *P. aeruginosa* biofilm formation (Bala et al., 2015). There are at least four QS systems in *P. aeruginosa*: *las*, *rhl*, *pqs*, and *iqs* (Lee and Zhang, 2015). As the two main QS systems of *P. aeruginosa*, both *las* and *rhl* systems use acyl homoserine lactone (AHL) as a signal molecule, which binds to the signal molecule receptor protein to play a regulatory role (Huang et al., 2019). The *las* system represses the *pel* locus, an operon encoding for the synthesis of extracellular matrix polysaccharides that induce biofilm formation and dispersion (Lin and Cheng, 2019), while the *rhl* system positively regulates the production of the biosurfactant rhamnolipid, which is important for late biofilm formation (Davey et al., 2003; Lequette and Greenberg, 2005; Chrzanowski et al., 2012). The *P. aeruginosa pqs* system uses 2-alkyl-4-quinolones (AQs) as signaling molecules. These AQs mainly include 2-heptyl-3-hydroxy-4-quinolone (PQS) and its precursor 2-heptyl-4-hydroxyquinoline (HHQ), both of which are recognized by the cognate receptor MvfR (Multiple virulence factor regulator, a *P. aeruginosa* quorum-sensing transcription factor, also known as PqsR; Lin et al., 2018a). Through interaction with this receptor, HHQ and PQS induce the expression of a variety of genes, including their own biosynthetic enzyme cascade and genes involved in biofilm formation (Schutz and Empting, 2018).

## Anti-biofilm strategies: Current approaches and perspectives

Antibiotics have been widely used to treat biofilm infections, but clinical treatment still faces many challenges due to drug resistance issues, biofilm matrices that hinder drug penetration, and drug-microbe interactions (Kamaruzzaman et al., 2018). Therefore, many new anti-biofilm technologies have been developed (Hughes and Webber, 2017), such as combining antibiotics and using new strategies, for example, gallium, phage therapy, and antimicrobial photodynamic therapy (aPDT), to inhibit biofilm formation. Table 1 summarizes the strategies discovered in recent years for the treatment of *P. aeruginosa* biofilm infection and the different action mechanisms of related anti-biofilm molecules.

**Table 1:** Different strategies to treat *Pseudomonas aeruginosa* infectious biofilms.

Strategy	Mechanism	Molecules associated	Reference
Antibiotics	Antibiotics are used in combination with antibiotics or other substances to destroy biofilms and prevent the development of antibiotic resistance	Gentamicin/ciprofloxacin, tobramycin/clarithromycin, linolenic acid-tobramycin, gentamicin-EDTA, glutamine-ampicillin	Chauhan et al., 2012; Chanda et al., 2017; Ghorbani et al., 2017; Wang et al., 2019; Zhao et al., 2021
AMPs^a^	Interact and penetrate with the bacterial cell membrane to cause the death of the bacteria	LL-37, P5, cationic peptide 1,037, MC1, WLBU2	Overhage et al., 2008; de la Fuente-Nunez et al., 2012; Lin et al., 2018b Martinez et al., 2019; Yu et al., 2019
QSIs^b^	Inhibit the QS system and interfere with signaling molecules and receptor proteins	Zingerone, trans-cinnamaldehyde and salicylic acid, chloroacetamide and maleimide analogs, halogenated furanone derivatives, M64, QSI4	Kumar et al., 2015; Ahmed et al., 2019; O'Brien et al., 2015; Maura and Rahme, 2017; Chang et al., 2019; Schutz et al., 2021
Enzymes or polysaccharides	Target extracellular polymeric substances (such as exopolysaccharides, matrix proteins, and eDNA) to disrupt biofilms	DNase I, glycoside hydrolases PelA and PslG, alginate lyase AlyP1400, A101, EPS273	Jiang et al., 2011; Hymes et al., 2013; Zhang et al., 2018; Daboor et al., 2021; Wu et al., 2021
Ga^3+^	Acts as a “Trojan horse” to disrupt bacterial Fe metabolism and inhibit *P. aeruginosa* growth	Desferrioxamine-gallium, Ga-Cit, Ga (NO_3_)_3_	Banin et al., 2008; Rzhepishevska et al., 2011; Kang et al., 2021
NO	Creates nitrosative stress or oxidative stress in the biofilm and aids in biofilm dispersal	NO-sensing proteins	Hossain and Boon, 2017
Bacteriophages	Encode enzymes to destroy the extracellular matrix	IME180, quorum quenching lactamase	Pei and Lamas-Samanamud, 2014; Mi et al., 2019
aPDT^c^	PS binds to the biofilm matrix and generates ROS under light, initiating multi-target damage	Tetra-Py^+^-Me, MB + GN, ICG-APTMS@SPION/laser	Beirao et al., 2014; Bilici et al., 2020; Perez-Laguna et al., 2020

a Antimicrobial peptides.

b Quorum-sensing inhibitors.

c Antimicrobial photodynamic therapy.

### Antibiotics

It is well known that antibiotic therapy is the most important and effective measure to control bacterial infection. However, bacterial biofilms are highly resistant to antibiotic treatment and immune response (Shrivastava et al., 2018; Srinivasan et al., 2021). Due to the low outer membrane permeability of *P. aeruginosa* and its own adaptive mechanisms, it is less susceptible to most antibiotics and readily achieves clinical resistance. The use of single antibiotics to treat *P. aeruginosa* biofilm infections therefore presents significant challenges, and various strategies have been developed to treat biofilms and prevent the development of antibiotic resistance, including increasing antibiotic concentrations or the use of antibiotics in combination (Khan et al., 2020). Here, we divide the mechanism of action of combined antibiotics into two main categories: the synergistic effect between different antibiotics and the combined use of other substances and antibiotics.

The combined use of different antibiotics against *P. aeruginosa* biofilms has been described in detail in a previous review (Yadav et al., 2021). The antibiotic combinations that have been found to be effective against *P. aeruginosa* biofilm include gentamicin/ciprofloxacin (Wang et al., 2019) and tobramycin/clarithromycin (Ghorbani et al., 2017). These combinations all increase the therapeutic efficacy of antibiotics against *P. aeruginosa* biofilm. In addition to the synergistic effect between antibiotics, substances such as metal chelators, fatty acids, and amino acids combined with antibiotics will also help to prevent the formation of *P. aeruginosa* biofilms. Linolenic acid (LNA) is an essential fatty acid that has antibacterial effects on various microorganisms. Studies have found that LNA can not only interfere with intercellular communication and reduce the production of virulence factors, but can also enhance the potency of tobramycin and synergistically inhibits biofilm formation by affecting *P. aeruginosa* quorum-sensing systems (Chanda et al., 2017). The cation chelator EDTA acts as a therapeutic adjuvant to destabilize biofilm matrices (Lebeaux et al., 2015). Some studies have found that the combination of EDTA and antibiotics can quickly and persistently remove biofilms formed *in vivo* compared with the use of antibiotics alone (Chauhan et al., 2012; Lebeaux et al., 2015). Glutamine is an amino acid that is used as a nutritional source, and the exogenous addition of glutamine can stimulate the influx of ampicillin, resulting in the accumulation of intracellular antibiotic concentrations that exceed the amount tolerated by multidrug-resistant bacteria. Glutamine-enhanced ampicillin-mediated killing has been found to be effective against *P. aeruginosa* biofilms in a mouse model of urinary tract infection. Moreover, glutamine also retards the development of ampicillin resistance, which may help in the future development of effective antibiotic drugs to prevent or manage difficult-to-treat bacterial biofilms (Zhao et al., 2021). In addition, the addition of extra O_2_ *via* hyperbaric oxygen therapy (HBOT) can increase the susceptibility of pathogens to several antibiotics against metabolically active bacteria by activating aerobic respiration. When combined with tobramycin or ciprofloxacin, re-oxygenation with HBOT enhanced the killing of clinical *P. aeruginosa* and the eradication of biofilms (Lichtenberg et al., 2022).

*In vitro* and *in vivo* experiments showed that the minimum inhibitory concentration (MIC) and minimum bactericidal concentration of biofilm bacterial cells were usually much higher than those of planktonic cells (by about 10–1,000 times; Hoiby et al., 2010, 2011). Therefore, it is difficult to achieve the eradication of biofilms *in vivo* with the use of conventional single antibiotics (Wu et al., 2015; Sharma et al., 2019), while the combination of antibiotics with different antibiotics or other substances to increase their effect as a new strategy for the treatment of biofilm infections has broad prospects. In addition to antibiotics, a variety of compounds have been clinically used to treat *P. aeruginosa* biofilm infection (Soren et al., 2020; Pang and Zhu, 2021). These treatment methods can be roughly divided into two categories: preventing biofilm formation and destroying formed biofilms. For example, antimicrobial peptides (AMPs) and quorum-sensing inhibitors (QSIs) can prevent biofilm formation by regulating the biofilm formation process, while some exopolysaccharide hydrolases and DNases can target biofilm matrix components to destroy biofilms.

### AMPs

The clinical application of AMPs is accelerating with increasing antibiotic resistance worldwide (Mahlapuu et al., 2016). AMPs are tiny macromolecules composed of amino acids that have the ability to stimulate innate immune responses and exhibit potent activity against a broad range of bacterial species, fungi, protozoa, and encapsulated viruses (Mahlapuu et al., 2016; Abdi et al., 2019; Seyfi et al., 2020). It is generally acknowledged that most AMPs can directly bind to the bacterial surface, such as the lipopolysaccharide (LPS) of Gram-negative bacteria, and then depolarize and permeate the membrane (Wen et al., 2013; Chou et al., 2019). LL-37, a classic human AMP, has been identified as capable of disrupting bacterial membranes, leading to cell death and inhibiting *P. aeruginosa* biofilm formation (Overhage et al., 2008), but subsequent study has shown that at sub-inhibitory concentrations it can promote *P. aeruginosa* DNA mutations and induce its resistance to antibiotics (Limoli et al., 2014). Despite this, as potential biofilm inhibitors, AMPs still hold great promise for the targeted elimination of biofilm proliferation in multi-drug resistant and extensively drug-resistant bacteria (Pontes et al., 2022). The target of these AMPs in the cell is typically the cell membrane. Through interacting with the bacterial cell membrane and penetrating the cell membrane, AMPs cause the death of the bacteria, thereby reducing the possibility of bacterial resistance (Annunziato and Costantino, 2020). As a promising class of compounds to overcome antimicrobial resistance, AMPs have been shown to have some advantages over traditional antibiotics.

Several studies have reported the mechanism of action of AMPs in detail (Annunziato and Costantino, 2020; Talapko and Skrlec, 2020). This review divides the anti-biofilm action mechanism of AMPs into two categories. AMPs in the first category exhibit anti-biofilm activity through their membrane dissolution mechanism, which directly affects the integrity of bacterial cell membranes and cell walls. The anti-biofilm peptide P5 has been found to have the ability to target the membrane permeability of *P. aeruginosa* and has synergistic and bactericidal effects with the carbapenem antibiotic meropenem (Martinez et al., 2019). An outer membrane permeability assay showed that P5 could easily permeate the cell membrane at concentrations below 0.5 × MIC, which occurred because meropenem entered the cytoplasmic space to interfere with the formation of peptidoglycans in the cell wall. In addition to meropenem, other antibiotics can also act on *P. aeruginosa* biofilms synergistically with AMPs, such as imipenem (Feng et al., 2015) and tobramycin (Beaudoin et al., 2018).

AMPs in the second category affect the growth pattern of biofilms by inhibiting bacterial adhesion and interfering with gene expression. While AMPs are generally considered to be membrane-active molecules that disrupt biofilms by perturbing the cell wall/membrane, AMPs also possess multifunctional activities such as protein synthesis and gene expression at multiple sites within the membrane or within the cell, enabling efficient killing (Le et al., 2017). A novel synthetic cationic peptide, 1,037, can significantly reduce *P. aeruginosa* biofilm formation and lead to cell death in biofilms at certain concentrations. Analysis of its effect on gene expression has revealed that 1,037 directly inhibits biofilms by reducing swimming and swarming motilities, stimulating twitching motility, and inhibiting the expression of various genes involved in biofilm formation, such as *PA2204* (de la Fuente-Nunez et al., 2012). Recent studies have found that the anti-biofilm peptide MC1 can inhibit biofilm formation by down-regulating the relative expression levels of *pelA*, *algD*, and *pslA* genes in *P. aeruginosa* and reducing the synthesis of exopolysaccharides (Yu et al., 2019). Another well-studied AMP, called WLBU2, and its D-amino acid enantiomer D8 have also shown gene modulating activity against *P. aeruginosa* in the biofilm mode of growth, as well as increased safety, stability, and antimicrobial efficacy when substituting the L-amino acids in WLBU2 with D-amino acids (Lashua et al., 2016; Lin et al., 2018b; Di et al., 2020).

As anti-biofilm peptides can inhibit the formation of biofilms or remove mature biofilms, they have been gradually recognized by an increasing number of researchers as a potential new drug for the prevention and treatment of bacterial biofilm infections. However, there are still many obstacles to clinical application. For example, anti-biofilm peptides tend to show a certain degree of hemolysis or cytotoxicity to eukaryotic cells, and they are easily hydrolyzed by protease and cannot exist stably *in vivo* (Aoki and Ueda, 2013). In addition, the high production cost of anti-biofilm peptides and long drug development cycle also limit their potential clinical application.

### QSIs

In recent years, an increasing number of studies have developed new antibacterial drugs by targeting specific virulence factors or their regulatory mechanisms to reduce the emergence of drug-resistant strains (Muhlen and Dersch, 2016; Kamal et al., 2017). One of these strategies is directed toward interference with QS-mediated signaling. The QS system of *P. aeruginosa* consists of four systems that interact to form a complex intercellular communication network that regulates the expression of its virulence-related genes and biofilm formation in a cell-density-dependent manner by generating QS signaling molecules (Rutherford and Bassler, 2012; Yong et al., 2018; Lin et al., 2018a).

QSIs can be either natural or synthetic, and both types of QSIs can inhibit the formation of biofilms by targeting different sites (Kalia, 2013). This review divides QSIs into two categories: QSIs that inhibit the expression of the QS system and QSIs that interfere with the combination between signaling molecules and receptor proteins. The first type of QSI interferes with and inhibits the expression of the QS system, altering the *P. aeruginosa* biofilm architecture and enabling antibiotics to better penetrate and more efficiently kill bacterial cells. In a previous study, the naturally isolated plant compounds trans-cinnamaldehyde and salicylic acid effectively down-regulated both the *las* and *rhl* QS systems, significantly inhibited the expression of QS regulatory and virulence genes in *P. aeruginosa* PAO1, and also reduced biofilm formation concomitantly with repressed rhamnolipid gene expression (Rajkumari et al., 2018; Ahmed et al., 2019). In addition, a host of synthetic biofilm inhibitors have also been developed. Chloroacetamide and maleimide analogs, as potent, drug-like small molecule inhibitors of QS in *P. aeruginosa*, are anticipated to be of significant medical interest. These inhibitors exhibit potent LasR antagonist activity and inhibit the expression of the *P. aeruginosa* virulence factor pyocyanin, as well as biofilm formation in PAO1 and PA14 (O'Brien et al., 2015). In addition, Chang et al. identified a new series of halogenated furanone derivatives and found that they effectively inhibited *lasB* expression in a dose-dependent manner and showed remarkable biofilm formation in *P. aeruginosa* (Chang et al., 2019).

The second category of QSI molecules functions by interfering with and inhibiting the combinations between signaling molecules and receptor proteins that are required for bacterial cell-to-cell communication, the production of virulence factors, and biofilm formation (Soukarieh et al., 2018). Ginger has been widely used as a medicinal herb with strong antimicrobial properties. Zingerone is one of the main components of dry ginger root, is found in many herbal spices, and can effectively regulate the biofilm structure of *P. aeruginosa* (Kumar et al., 2013). In *P. aeruginosa*, zingerone inhibits the *las*, *rhl*, and *pqs* systems by binding to their respective cognate receptors (LasR, RhlR, and MvfR), which silences the cell communication network and ultimately suppresses the virulence and biofilm formation of *P. aeruginosa* (Kumar et al., 2015). Furthermore, MvfR, as a crucial transcriptional regulator of the PQS system of *P. aeruginosa*, is considered to a potential target for inhibiting the PQS-MvfR QS system (Kitao et al., 2018). The benzamide-benzimidazole compound M64 can inhibit the *P. aeruginosa* QS regulator MvfR, resulting in reduced biofilm formation and the increased susceptibility of *P. aeruginosa* to antibiotics (Maura and Rahme, 2017). Recently, a study has reported a novel class of QSIs called QSI4, which possesses excellent activity in inhibiting pyocyanin production and the MvfR reporter-gene; when combined with antibiotics, QSI4 has a significant synergistic effect on the elimination of *P. aeruginosa* biofilm (Schutz et al., 2021).

As an important intercellular communication system in *P. aeruginosa*, the QS system plays a key role in the regulation of biofilm formation. QSIs inhibit biofilm formation through anti-virulence or a pathoblocker approach, which can synergize the efficacy of antibiotics but does not affect the viability of bacteria. Clinical application of QSIs will reduce the development of antibiotic resistance as well as some toxic side effects (Wagner et al., 2016). Therefore, QSIs are currently promising drug targets for the prevention and treatment of *P. aeruginosa* infection.

### Targeting polysaccharides

As functionally rich and dynamically changing communities, biofilms can modify matrix components to adapt to changes in various environmental conditions and pressures. In *P. aeruginosa*, enzymes targeting biofilm EPSs may offer a general strategy to prevent clinical biofilm infections (Zhao et al., 2018). The bacteria themselves also synthesize polysaccharides or certain endogenous matrix-degrading enzymes to induce the dispersion of the biofilm, such as glycoside hydrolases (Srinivasan et al., 2021).

**Figure 2:**
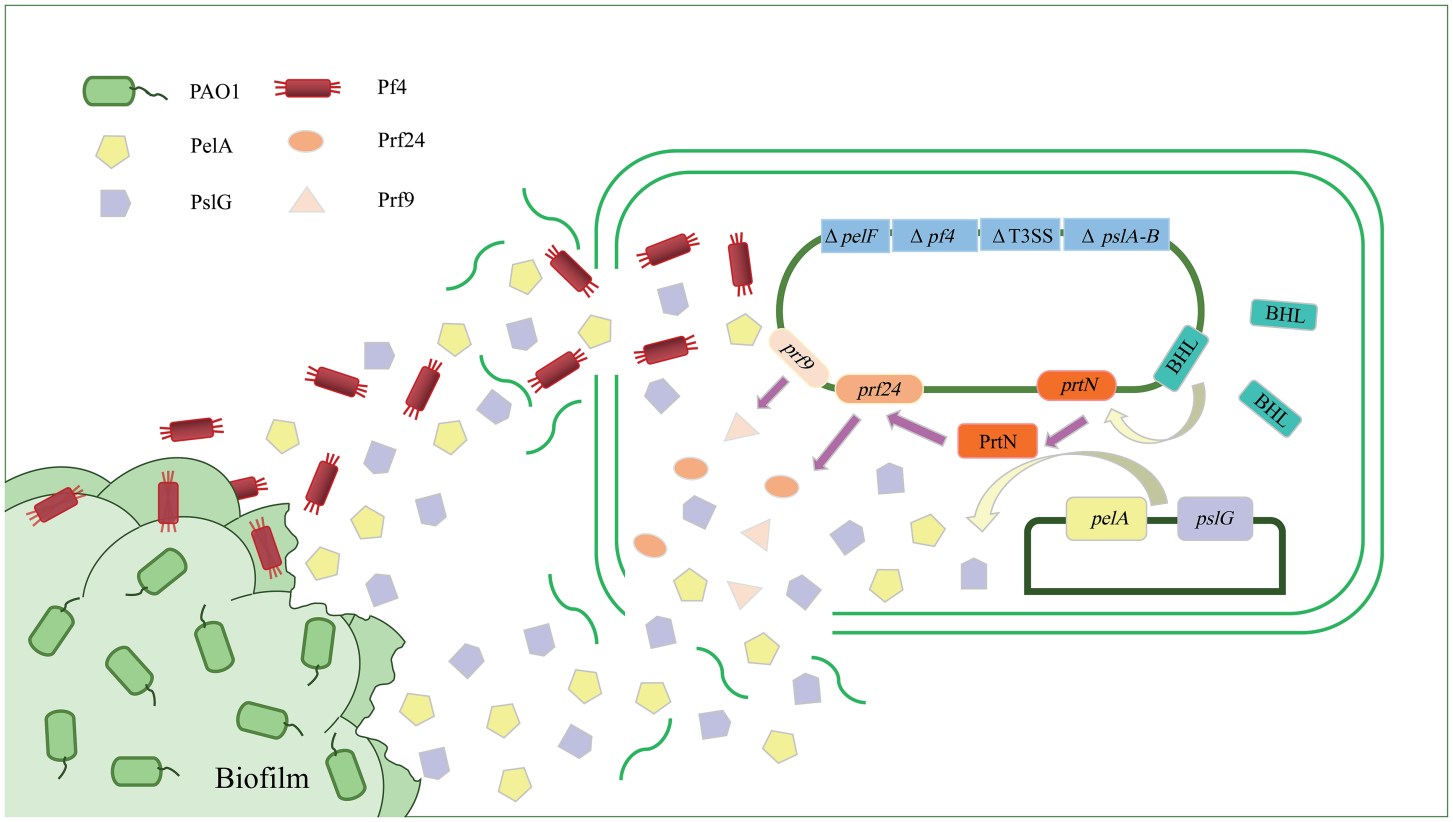
Schematic diagram of targeted delivery of extracellular glycoside hydrolase by engineered bacteria to destroy *Pseudomonas aeruginosa* biofilm. The biofilm formation and virulence-related genes *pelA-B*, *pelF*, and T3SS of *P. aeruginosa* were knocked out as parental strains, and exogenous recombinant vectors were introduced to overexpress the exopolysaccharide hydrolases PelA and PslG. PelA and PslG accumulated in cells and were then released into the extracellular matrix through cell lysis. There are two ways in which hydrolases are released into the extracellular matrix. The first is through regulating the *prtN* gene to activate the expression of cell lysis protein genes, thereby releasing PelA and PslG. The second is by deleting the Pf4 filamentous prophage-encoding gene cluster to sensitize it to the Pf4 phage in biofilms, thereby initiating the passive lysis mechanism of its own cells to release PelA and PslG. The PelA and PslG are released to the extracellular matrix to destroy the biofilm skeleton components Pel and Psl through enzymatic hydrolysis, thereby destroying the *P. aeruginosa* biofilm.

A key component of biofilm formation is the biosynthesis of the exopolysaccharides Pel, Psl, and alginate (Limoli et al., 2015). Enzymes targeting the extracellular matrix could serve as targets to disrupt biofilms. Alginate lyase can degrade alginate through the β-elimination of glycosidic bonds to disrupt the structure and integrity of biofilms and significantly increase biofilm diffusion (Kovach et al., 2020). Recently, a study has reported an alginate lyase (AlyP1400) purified from a marine bacterial *Pseudoalteromonas* species that can treat *P. aeruginosa* infections in cystic fibrosis lungs or other *P. aeruginosa* biofilm-related infections by combining the use of the alginate lyase with antibiotics (Daboor et al., 2021). The glycoside hydrolases alpha-amylase and cellulase can also break down complex polysaccharides, convert the bacteria into a planktonic state, effectively destroy *P. aeruginosa* biofilm, and increase antibiotic efficacy (Fleming et al., 2017; Redman et al., 2020). In addition, PelA and PslG, as naturally derived glycoside hydrolases, can selectively target and degrade exopolysaccharides in the biofilm matrix, thus destroying the biofilm (Baker et al., 2016; Zhang et al., 2018). In a previous study, overexpressed or exogenously supplied PslG prevented biofilm formation by degrading Psl (Yu et al., 2015). As a hydrolase, PelA can scavenge polysaccharides in the periplasm, and its deacetylase activity is related to the formation of biofilms and the morphology of wrinkled colonies (Colvin et al., 2013; Marmont et al., 2017). Recently, our team has used *P. aeruginosa* as the starting strain to construct an engineered bacterium for the targeted transport and delivery of functional proteins that can use two polysaccharide hydrolases, PelA and PslG, to target biofilms (Figure 2). First, the engineered bacterium was constructed through synthetic biology so that it could initiate the lysis of its own cells, and then, the recombinant vectors were introduced to overexpress the two exopolysaccharide hydrolase genes *pelA* and *pslG*. Finally, the effect of engineered bacteria on *P. aeruginosa* biofilm was detected by biofilm formation experiments. It was found that overexpression of *pelA* and *pslG* could accumulate polysaccharide hydrolases in the intracellular matrix and release them to the extracellular matrix through the cell lysis site to disrupt the biofilm cytoskeletal components Psl and Pel, eventually destroying the biofilm and preventing further biofilm formation (Wang et al., 2021).

The extracellular biofilm matrix of *P. aeruginosa* is mainly composed of exopolysaccharides, which are involved in the formation and maintenance of the structural biofilm (Franklin et al., 2011). However, some bacterial exopolysaccharides can perform functions that inhibit or destabilize the biofilm (Rendueles et al., 2013). A former study showed that *P. aeruginosa* extracellular products, mainly polysaccharides, disrupted established biofilms (Qin et al., 2009). Recently, more exopolysaccharides have been found to show negative activity against biofilm formation in *P. aeruginosa*. A bacterial exopolysaccharide (A101) not only inhibits the biofilm formation of many bacteria but also disrupts established biofilms. In addition, A101 increased the ability of aminoglycoside antibiotics to eliminate *P. aeruginosa* biofilm, which may indicate that A101 has potential in the design of new therapeutic strategies for bacterial biofilm-associated infections and in limiting biofilm formation on medical indwelling devices (Jiang et al., 2011). A recent experiment has reported an exopolysaccharide, EPS273, that reduces biofilm formation in *P. aeruginosa* by reducing the expression levels of the two-component system *phoP*–*phoQ*, which then regulates the expression levels of the QS systems *lasI/lasR* and *rhlI/rhlR*. The QS system further regulates the genes involved in biofilm formation, such as the genes involved in the production of the extracellular matrix and virulence factors, genes involved in flagella and cell motility, and genes involved in iron acquisition (Wu et al., 2021).

### DNase

eDNA plays a structural role in the formation of biofilms (Allesen-Holm et al., 2006). eDNA can serve as a source of nutrients for bacteria under starvation and is involved in facilitating the twitching motility-mediated expansion of biofilms (Finkel and Kolter, 2001; Wei and Ma, 2013). In addition, eDNA interacts with Psl to form eDNA-Psl to provide structural support for biofilms (Wang et al., 2015). Based on these factors, drug pathways for targeting biofilm matrices *via* eDNA are emerging.

Deoxyribonuclease I (DNase I) is the only enzyme used clinically to disrupt the biofilm of *P. aeruginosa*. This therapeutic enzyme disrupts biofilms through the hydrolysis of DNA in the extracellular matrix (Hymes et al., 2013). DNase is involved in breaking the phosphodiester linkage in the eDNA molecular backbone in biofilms. As eDNA is essential for the initial attachment and aggregation of EPSs on the surface, this makes it difficult for bacteria to form an intact biofilm (Das et al., 2010; Srinivasan et al., 2021). Immature *P. aeruginosa* biofilms are therefore more sensitive to DNase treatment than mature biofilms (Hymes et al., 2013). Some studies have found that L-methionine (L-Met) can destroy *P. aeruginosa* biofilms through up-regulating DNase genes and inducing the expression of DNase, thereby targeting eDNA in biofilms (Gnanadhas et al., 2015). In addition, some DNase-like proteins have also been found to prevent and destroy bacterial biofilms. DNase1-like 2 (DNase1L2) expressed in human stratum corneum has enzymatic activity, can degrade eDNA, and can effectively inhibit *P. aeruginosa* biofilm formation (Eckhart et al., 2007). In addition, DNase I coatings are used as antimicrobial coatings for modern medical equipment. DNase Ι immobilization on surfaces has shown promise in reducing bacterial adhesion to surfaces, as this enzyme targets single biofilms and can effectively cleave eDNA on bacterial cell surfaces that are essential for bacterial adhesion (Swartjes et al., 2013; Sharma and Pagedar Singh, 2018). With the ongoing rise in antibiotic resistance, DNase Ι coating may provide a timely, potent new approach to prevent the formation of biofilms on biomaterial implants and devices.

When the biofilm matures to a certain extent, DNase treatment is no longer effective (Whitchurch et al., 2002). This resistance to DNase may be due to the replacement or supplementation of eDNA by other extracellular matrix components, or the binding of eDNA to another component that protects it from enzymatic degradation (Okshevsky et al., 2015). Therefore, enzymatic hydrolysis methods that destabilize biofilms by enzymatically degrading eDNA should also target polysaccharides or proteins bound to eDNA. For example, the interactions between eDNA with Psl and Type IV pili play important roles in biofilm formation (Barken et al., 2008), and disrupting these interactions could also be a potentially interesting target for biofilm treatment. In addition to DNase, the accumulation of eDNA itself in biofilms and infection sites can acidify the local environment (Wilton et al., 2016). The acidic environment stimulated increased *P. aeruginosa* biofilm formation, promoted faster bacterial evolution to improve antibiotic resistance, and increased the expression of multiple biofilm/virulence-related genes. Therefore, the use of simple pH-buffering agents alongside antibiotics may be a novel treatment strategy for combating chronic infection in the acidic, DNA-enriched lungs of clinic patients (Lin et al., 2021).

### Gallium (Ga)

Recently, Ga ions have shown excellent anti-*Pseudomonal* activity and have been used as a novel biofilm treatment approach (Smith et al., 2017; Tummler, 2019; Tovar-Garcia et al., 2020). Previous research has used Ga as a “Trojan horse” to disrupt bacterial iron metabolism and exploit the Fe stress of *in vivo* environments because Ga has an ionic radius nearly identical to that of Fe, and many biological systems are unable to distinguish Ga^3+^ from Fe^3+^ (Kaneko et al., 2007). While Ga^3+^ chemically resembles Fe^3+^, unlike iron, Ga cannot be reduced under physiological conditions, which also inhibits some of its basic functions (Minandri et al., 2014). Iron is not only a necessary element for growth but is also a cue in biofilm formation (Banin et al., 2005). Thus, interfering with bacterial iron homeostasis may serve as a potential therapeutic target that can block *P. aeruginosa* virulence in both the free-living and biofilm states.

Previous research found that low concentrations of Ga inhibited *P. aeruginosa* growth and prevented biofilm formation. This inhibition was mediated by the repression of the transcriptional regulator *pvdS*, as overriding *pvdS* repression partially protected bacteria from the growth-inhibitory action of Ga (Kaneko et al., 2007). Besides Ga alone, some complexes with Ga can also target *P. aeruginosa* iron metabolism to inhibit biofilm formation, such as desferrioxamine-Ga (DFO-Ga) and Ga citrate (Ga-Cit). Desferrioxamine (DFO) is a hydroxamate-based siderophore that induces proteins related to iron citrate and iron DFO uptake in iron-starved *P. aeruginosa* (Llamas et al., 2006; Marshall et al., 2009). The complex DFO-Ga, which can kill free-living bacteria and prevent biofilm formation, has been approved for clinical use (Banin et al., 2008). However, recent research indicates that Ga-Cit has an anti-biofilm effect and is more bactericidal than Ga-DFO (Rzhepishevska et al., 2011). Like other Ga complexes, Ga-Cit significantly inhibited biofilm production in *P. aeruginosa* at concentrations as low as 10 μM (Rzhepishevska et al., 2011). Moreover, several studies have explored the activity of Ga in combination with antibiotics in search of useful synergistic effects. Ga nitrate has been found to be an effective antimicrobial agent that inhibits *P. aeruginosa* growth. A recent study found that Ga(NO_3_)_3_ and tetracycline alone had a bactericidal effect, and the combined use of the two strongly inhibited the formation of *P. aeruginosa* biofilm (Kang et al., 2021).

Ga^3+^ is effective in destroying bacterial biofilms, and many drugs based on Ga have been approved for clinical treatment with remarkable results (Goss et al., 2018). For example, Ga(NO_3_)_3_, which is approved by the US Food and Drug Administration (FDA) for the treatment of infections, can be used to treat both acute and chronic pneumonia caused by *P. aeruginosa* infection (Bonchi et al., 2015). In addition to Ga and its compounds, combined use with antibiotics has achieved remarkable results in clinical treatment. Therefore, Ga, as a target for destroying biofilms, has potential in the development of more effective drugs for the treatment of biofilm infections.

### Nitric oxide

*Pseudomonas aeruginosa* is a facultative anaerobe that can breathe under anaerobic conditions and denitrify in the presence of nitrate and nitrite. These abilities are associated with the virulence of bacteria (Cutruzzola and Frankenberg-Dinkel, 2016). NO is a radical diatomic gas molecule that plays important signaling roles in both eukaryotes and bacteria at low concentrations (Arora et al., 2015). NO has also been demonstrated to be an effective *P. aeruginosa* biofilm disruption agent that creates nitrosative or oxidative stress in the biofilm and induces the dispersal of *P. aeruginosa* and other bacterial biofilms by reducing c-di-GMP levels (Williams and Boon, 2019).

An early study of the effect of NO on biofilm formation showed that nanomolar NO caused biofilm dispersal in *P. aeruginosa* and enhanced the efficacy of antimicrobial compounds when combined with antibiotics (Barraud et al., 2006). This finding was confirmed in the clinical treatment of cystic fibrosis patients (Howlin et al., 2017). However, another study found that the exogenous addition of high concentrations of iron inhibited the diffusion of NO-induced biofilms and promoted the rapid attachment of plankton cells and resumed diffusion after the addition of iron chelator agents (Zhu et al., 2019). This is not due to the scavenging of NO by free iron but was related to an iron-induced cellular response that led to the increased production of the exopolysaccharide Psl and restored *P. aeruginosa* biofilm diffusion. Very recently, a novel family of heme-based NO binding proteins termed NO-sensing proteins (NosP) have been discovered in *P. aeruginosa* in the same operon as *PA1976* (NahK). NahK has been identified as a NosP-associated histidine kinase, and it has been previously associated with biofilm regulation (Hossain and Boon, 2017). Experiments have suggested that NosP binds to NO, which controls the phosphorylation of a histidine-containing phosphotransfer domain, thus resulting in biofilm dispersal. However, the specific players involved in the signaling pathway have yet to be identified.

Although NO has attracted particular interest due to its role in biofilm dispersal, this approach still presents many practical issues in clinical trials. For example, the current inhaled NO therapy method is extremely expensive, largely owing to the difficulty in handling the gas and its incompatibility with oxygen, which results in the formation of toxic nitrogen dioxide (NO_2_; Yang et al., 2015). Therefore, while the bactericidal effect of antibacterial agents on biofilm-infected sites can be enhanced in a targeted manner, the NO-mediated toxicity should also be reduced so that it can be used in clinical anti-biofilm therapy (Barraud et al., 2012). In addition, the use of NO in combination with antibiotics can enhance the NO-mediated bactericidal effect and improve the specificity of NO delivery, so the use of NO is still very promising (Poh and Rice, 2022).

### Bacteriophage therapy

Bacteriophages are natural bacterial viruses (Clokie et al., 2011). As they are unaffected by antibiotic resistance, bacteriophages have been used as therapeutic agents in early clinical practice (Kutter et al., 2010; Abedon et al., 2011). With the emergence of antibiotic-resistant strains, phage therapy has once again drawn attention, and a growing body of research has validated the use of bacteriophages in therapy and prophylaxis in the fight against drug-resistant bacteria (Kutateladze and Adamia, 2010). Phages can encode enzymes that degrade polymers and inhibit *P. aeruginosa* biofilm formation by disrupting the extracellular matrix and increasing the permeability, allowing antibiotics to reach the inner layer of biofilms (Harper et al., 2014). Therefore, an increasing number of studies have used bacteriophages to develop drugs to treat biofilm infections (Soothill, 2013).

The pathways by which phages destroy biofilms can be divided into two categories. First, phages can destroy biofilm structures by inducing the synthesis of enzymes such as polysaccharide depolymerases in *P. aeruginosa* (Pires et al., 2016; Chegini et al., 2020). Polysaccharide depolymerase is a polysaccharide hydrolase encoded by bacteriophages that can specifically degrade macromolecular carbohydrates on the host bacterial envelope (Yan et al., 2014). IME180, a *P. aeruginosa* phage isolated from a hospital, encodes an exopolysaccharide-degrading enzyme that is highly homologous to deglycans and can effectively degrade exopolysaccharides, inhibiting the formation of host bacterial biofilms and destroying established biofilms (Mi et al., 2019). In addition to polysaccharide hydrolases, bacteriophages can also produce endolysins, which inhibit bacterial cell wall synthesis by hydrolyzing peptidoglycan (Schmelcher et al., 2012). Another way in which bacteriophages inhibit biofilms is through the production of enzymes that inhibit biofilm production. A study has reported that phages can be genetically modified to induce the synthesis of quorum quenching lactamase, thereby inhibiting bacterial biofilm formation (Pei and Lamas-Samanamud, 2014). An engineered T7 phage incorporating the AHL lactonase *aiiA* gene can hydrolyze acyl AHL and inhibit QS activities in *P. aeruginosa*, ultimately inhibiting biofilm production (Whiteley et al., 2017). Unlike polysaccharide depolymerases, which can degrade one or several related polysaccharides, the T7*aiiA* phage can affect multiple bacteria in mixed-strain biofilms, rather than the host bacteria alone.

In addition to being directly used as a tool to destroy biofilms, phages can also indirectly aid in other strategies to destroy biofilms. In *P. aeruginosa* PAO1, Pf4 filamentous phages naturally parasitize by integrating into the genome and play critical roles in PAO1 virulence, biofilm development, and structural stability. Studies have shown that *P. aeruginosa* lacking the Pf4 filamentous phage-encoding gene cluster is highly susceptible to Pf4 filamentous phages. Therefore, we mutated the gene cluster encoding Pf4 filamentous phage when constructing the engineered bacteria, which made it very sensitive to Pf4 filamentous phage in biofilms. Upon contact, Pf4 filamentous phage could infect and lyse the engineered bacteria to release the intracellular accumulation of exopolysaccharide hydrolase PelA and PslG, thereby destroying the biofilm of *P. aeruginosa* (Figure 2; Wang et al., 2021).

As mentioned earlier, bacteriophages are considered to be potential drugs for the prevention and control of biofilms due to their infection diversity and specificity (Melo et al., 2019). However, the application of phages in biofilm control still has some limitations (Abedon et al., 2021). For example, a reduction in the metabolic activity of biofilm bacterial cells due to phage infection is dependent on host growth conditions (Chegini et al., 2020) and stimulates the rapid release of bacterial endotoxins, leading to an inflammatory response (Ferriol-Gonzalez and Domingo-Calap, 2020).

### aPDT

aPDT is an emerging non-invasive treatment method that uses non-toxic photosensitizer (PS), specific wavelengths of visible or near-infrared light, and molecular oxygen around or inside pathogens to produce phototoxic reactions to kill pathogens (Rajesh et al., 2011). aPDT can also destroy microbial biofilms in a process that consists of two steps. The first step is the binding of PSs to the biofilm matrix. Although some types of PSs only bind to the cell surface, most types of PSs can pass through the cytoplasmic membrane and enter the cytoplasm. PSs bound to the biofilm matrix generate reactive oxygen species (ROS) under light, thereby initiating multi-target damage (Hu et al., 2018), which attacks various biofilm components, leading to disintegration, including the disintegration of lipids, proteins, DNA, and exopolysaccharides in the matrix (Dosselli et al., 2012; Martins Antunes de Melo et al., 2021). Studies have reported many examples of aPDT used in the treatment of biofilm infections, and some have been used in clinical trials (Tahmassebi et al., 2015; Liang et al., 2020).

Polysaccharides are the most abundant polymers in biofilm matrices In the presence of a certain concentration of Tetra-Py^+^-Me, the polysaccharide concentration in the *P. aeruginosa* biofilm was significantly reduced after irradiation, and its biofilm substrate was attacked by photodynamic force and destroyed (Beirao et al., 2014). aPDT targeting related proteins also affect *P. aeruginosa* biofilm formation. The PS methylene blue (MB) combined with the antibiotic gentamicin (GN) has a synergistic antibacterial effect on plankton. Adding GN at a concentration where MB alone does not have a significant antibacterial effect can exert a positive bactericidal effect against *P. aeruginosa* biofilms. This synergistic killing mechanism may be caused by GN acting on the level of protein synthesis, changing the permeability of the bacterial wall and thereby promoting the accumulation of MB, but its potential mechanism needs further research (Perez-Laguna et al., 2020). Recently, antimicrobial photothermal therapy (aPTT) was demonstrated to be a promising method to eliminate planktonic cells and biofilms (Al-Bakri and Mahmoud, 2019; Li et al., 2019). The combined use of aPDT and aPTT has also become an effective local replacement therapy for the treatment of antibiotic-resistant bacterial infections and biofilms (Bilici et al., 2020). While 3-aminopropylsilane-coated superparamagnetic iron oxide nanoparticles have no significant inhibition on biofilms without laser treatment, the addition of laser treatment significantly reduces *P. aeruginosa* biofilms. Furthermore, after combining nanoparticles with PS, the biofilm can be reduced again. This combination of nanoparticles and PS may enhance the treatment of drug-resistant bacteria and their biofilms through the dual aPDT/aPTT mechanism (Bilici et al., 2020).

Due to its multi-targeted damage to microbial cells and its inability to induce drug resistance, aPDT has received increasing attention as an alternative treatment (Cieplik et al., 2018), and it is also effective in combination with antibiotics (Feng et al., 2021). However, the application of aPDT has certain limitations. The limitation of light transmission conditions makes it more effective in the clinical treatment of local infections. In addition, the characteristics of PS and the corresponding light source affect its application. PS with low molecular weight and high penetrating power should be selected, and the cost should also be controlled (Wainwright et al., 2017).

## Conclusions and prospective applications

*Pseudomonas aeruginosa* biofilm includes three main parts: exopolysaccharides, eDNA, and matrix protein. Different components play different roles in its adhesion, maturation, and dispersal processes and are regulated by factors such as the QS system. The most commonly used treatment of *P. aeruginosa* biofilm infection is mainly a single antibiotic treatment, but this clinical treatment faces many challenges due to drug resistance. With the development of drug-resistant strains, many promising therapeutic strategies have been developed to address these issues, such as combining antibiotics, targeting biofilms through enzymes or quorum-sensing systems, or using new photodynamic therapies and other compounds to prevent or inhibit *P. aeruginosa* biofilm formation by targeting the diffusion of biofilm formation. NO has also been shown to regulate biofilms through targeted diffusion (McDougald et al., 2011), exerting an inhibitory effect in the final stages of biofilm formation. However, when using dispersion as a therapeutic strategy, it is uncertain how efficient the dispersion reaction needs to be to become an effective therapeutic agent, and clinical treatments are mostly conducted on mixed biofilms (Lee and Yoon, 2017), which requires researchers to further explore diffusing agents and diffusion-inducing agents.

In addition to the above-mentioned therapeutic strategies, there are many other interesting methods that can be used to remove *P. aeruginosa* biofilms in clinical practice. *P. aeruginosa* itself can produce signal molecules that have inhibitory activity on its formed biofilm. For example, cis-2-decenoic acid, a short-chain fatty acid produced by *P. aeruginosa*, acts as a dispersal signal targeting the biofilm of some bacteria (Rahmani-Badi et al., 2014; Vuotto and Donelli, 2019; Jiang et al., 2020). In addition, some new materials such as nanoparticles, a class of emerging antibacterial agents, exhibit an antibacterial mechanism that includes the destruction of bacterial biofilms, and many innovative anti-biofilm nanomedicines and nanomaterials have been developed for clinical treatment (Xiu et al., 2021). A recent study developed a novel photocatalytic quantum dot-armed bacteriophage nanosystem that combined phage therapy and photodynamic therapy, not only specifically binding to host *P. aeruginosa*, but also targeting host bacteria through the inherent infectivity of phages, locally generating massive amounts of ROS under visible light irradiation, and thereby demonstrating potent anti-biofilm activity (Wang et al., 2022). However, microorganisms adhere to the surfaces of medical devices and are prone to forming biofilms, leading to inevitable and challenging issues with *P. aeruginosa* biofilm infection caused by the use of clinical medical devices (Wi and Patel, 2018). The surface modification of biomaterials has been the focus of extensive research to decrease microbial colonization and biofilm formation, and has been reviewed in detail (Yadav et al., 2021). An effective antimicrobial surface coating can prevent *P. aeruginosa* from adhering, achieving an anti-biofilm effect. One study achieved zero *P. aeruginosa* biofilm adhesion by adding lubricating fluids, consisting of perfluorinated liquids, to porous polytetrafluorethylene (PTFE) to fabricate liquid-infused surfaces (Epstein et al., 2012). In addition, the production of *P. aeruginosa* biofilms was reduced by four orders of magnitude after using a slippery omniphobic covalently attached liquid surface compared to polydimethylsiloxane (PDMS), a widely used medical implant material (Zhu et al., 2022). This biofilm-resistant liquid-like solid surface provides a novel strategy for the treatment of *P. aeruginosa* biofilms.

In addition to *P. aeruginosa*, there are multiple microorganisms that cause biofilm-related diseases, such as *Staphylococcus aureu*s, *Candida albicans*, and *Mycobacterium tuberculosis*, which cause serious global health problems due to their resistance to antimicrobial agents. The rapid development of new antimicrobial agents to overcome resistance is urgent, and gaining insights into the specific mechanisms of biofilm occurrence and their interactions with the host is key to solving the problem. Although biofilms have been studied through genomics, proteomics, and RNA sequencing, the rapid evolution of microorganisms has exceeded the pace of therapeutic technology development. New technologies to monitor biofilm formation and the responses of biofilm to antibiotic therapy are required. Furthermore, direct eradication becomes difficult as pathogens evolve defenses against antimicrobial agents, and inhibiting bacterial virulence may be more effective than killing bacteria while also providing new possibilities for the treatment of biofilm infections.

## Author contributions

JL conceptualized the article and critically revised the work. RY, JC, JW, and PL performed the literature search and wrote the manuscript. RY and JC prepared the figures and tables. All authors contributed to the article and approved the submitted version.

## Funding

This work was supported by the National Natural Science Foundation of China (32070103, 31860012, and 31700031), the Natural Science Basic Research Plan in Shaanxi Province of China (2021JM-415), the Regional Development Talent Project of the “Special Support Plan” of Shaanxi Province, a grant from the Outstanding Young Talent Support Plan of the Higher Education Institutions of Shaanxi Province, the Youth Innovation Team of Shaanxi Universities (2022), and by the Startup Foundation for Doctors of Yan’an University (YDBK2016-01).

## Conflict of interest

The authors declare that the research was conducted in the absence of any commercial or financial relationships that could be construed as a potential conflict of interest.

## Publisher’s note

All claims expressed in this article are solely those of the authors and do not necessarily represent those of their affiliated organizations, or those of the publisher, the editors and the reviewers. Any product that may be evaluated in this article, or claim that may be made by its manufacturer, is not guaranteed or endorsed by the publisher.

## References

[ref1] Abdi M. Mirkalantari S. Amirmozafari N. (2019). Bacterial resistance to antimicrobial peptides. J. Pept. Sci. 25:e3210. doi: 10.1002/psc.3210 31637796

[ref2] Abedon S. T. Danis-Wlodarczyk K. M. Wozniak D. J. Sullivan M. B. (2021). Improving phage-biofilm in vitro experimentation. Viruses 13, 1175. doi: 10.3390/v13061175, PMID: 34205417 PMC8234374

[ref3] Abedon S. T. Kuhl S. J. Blasdel B. G. Kutter E. M. (2011). Phage treatment of human infections. Bacteriophage 1, 66–85. doi: 10.4161/bact.1.2.15845, PMID: 22334863 PMC3278644

[ref4] Ahmed S. Rudden M. Smyth T. J. Dooley J. S. G. Marchant R. Banat I. M. (2019). Natural quorum sensing inhibitors effectively downregulate gene expression of *Pseudomonas aeruginosa* virulence factors. Appl. Microbiol. Biotechnol. 103, 3521–3535. doi: 10.1007/s00253-019-09618-0, PMID: 30852658 PMC6449319

[ref5] Al-Bakri A. G. Mahmoud N. N. (2019). Photothermal-induced antibacterial activity of gold nanorods loaded into polymeric hydrogel against *Pseudomonas aeruginosa* biofilm. Molecules 24, 2661. doi: 10.3390/molecules24142661, PMID: 31340472 PMC6680386

[ref6] Al-Dahmoshi H. Al-Obaidi R. Al-Khafaji N. (2021). “*Pseudomonas aeruginosa*: diseases, biofilm and antibiotic resistance.” In: *Pseudomonas aeruginosa* — Biofilm Formation, Infections and Treatments. eds. Das T. (London: InTechOpen Limited).

[ref7] Allesen-Holm M. Barken K. B. Yang L. Klausen M. Webb J. S. Kjelleberg S.. (2006). A characterization of DNA release in *Pseudomonas aeruginosa* cultures and biofilms. Mol. Microbiol. 59, 1114–1128. doi: 10.1111/j.1365-2958.2005.05008.x, PMID: 16430688

[ref8] Annunziato G. Costantino G. (2020). Antimicrobial peptides (AMPs): A patent review (2015-2020). Expert Opin. Ther. Pat. 30, 931–947. doi: 10.1080/13543776.2020.1851679, PMID: 33187458

[ref9] Martins Antunes de Melo W. C. Celiesiute-Germaniene R. Simonis P. Stirke A. (2021). Antimicrobial photodynamic therapy (aPDT) for biofilm treatments. Possible synergy between aPDT and pulsed electric fields. Virulence 12, 2247–2272. doi: 10.1080/21505594.2021.1960105 34496717 PMC8437467

[ref10] Aoki W. Ueda M. (2013). Characterization of antimicrobial peptides toward the development of novel antibiotics. Pharmaceuticals (Basel) 6, 1055–1081. doi: 10.3390/ph6081055, PMID: 24276381 PMC3817730

[ref11] Arora D. P. Hossain S. Xu Y. Boon E. M. (2015). Nitric oxide regulation of bacterial biofilms. Biochemistry 54, 3717–3728. doi: 10.1021/bi501476n 25996573

[ref12] Azam M. W. Khan A. U. (2019). Updates on the pathogenicity status of *Pseudomonas aeruginosa*. Drug Discov. Today 24, 350–359. doi: 10.1016/j.drudis.2018.07.003, PMID: 30036575

[ref13] Baker P. Hill P. J. Snarr B. D. Alnabelseya N. Pestrak M. J. Lee M. J.. (2016). Exopolysaccharide biosynthetic glycoside hydrolases can be utilized to disrupt and prevent *Pseudomonas aeruginosa* biofilms. Sci. Adv. 2:e1501632. doi: 10.1126/sciadv.1501632, PMID: 27386527 PMC4928890

[ref14] Bala A. Kumar L. Chhibber S. Harjai K. (2015). Augmentation of virulence related traits of *pqs* mutants by *pseudomonas* quinolone signal through membrane vesicles. J. Basic Microbiol. 55, 566–578. doi: 10.1002/jobm.201400377, PMID: 25283438

[ref15] Banin E. Lozinski A. Brady K. M. Berenshtein E. Butterfield P. W. Moshe M.. (2008). The potential of desferrioxamine-gallium as an anti-*pseudomonas* therapeutic agent. Proc. Natl. Acad. Sci. U. S. A. 105, 16761–16766. doi: 10.1073/pnas.0808608105, PMID: 18931304 PMC2575493

[ref16] Banin E. Vasil M. L. Greenberg E. P. (2005). Iron and *Pseudomonas aeruginosa* biofilm formation. Proc. Natl. Acad. Sci. U. S. A. 102, 11076–11081. doi: 10.1073/pnas.0504266102, PMID: 16043697 PMC1182440

[ref17] Barken K. B. Pamp S. J. Yang L. Gjermansen M. Bertrand J. J. Klausen M.. (2008). Roles of type IV pili, flagellum-mediated motility and extracellular DNA in the formation of mature multicellular structures in *Pseudomonas aeruginosa* biofilms. Environ. Microbiol. 10, 2331–2343. doi: 10.1111/j.1462-2920.2008.01658.x, PMID: 18485000

[ref18] Barraud N. Hassett D. J. Hwang S. H. Rice S. A. Kjelleberg S. Webb J. S. (2006). Involvement of nitric oxide in biofilm dispersal of *Pseudomonas aeruginosa*. J. Bacteriol. 188, 7344–7353. doi: 10.1128/JB.00779-06, PMID: 17050922 PMC1636254

[ref19] Barraud N. Kardak B. G. Yepuri N. R. Howlin R. P. Webb J. S. Faust S. N.. (2012). Cephalosporin-3'-diazeniumdiolates: targeted NO-donor prodrugs for dispersing bacterial biofilms. Angew. Chem. Int. Ed. Engl. 51, 9057–9060. doi: 10.1002/anie.201202414, PMID: 22890975

[ref20] Beaudoin T. Stone T. A. Glibowicka M. Adams C. Yau Y. Ahmadi S.. (2018). Activity of a novel antimicrobial peptide against *Pseudomonas aeruginosa* biofilms. Sci. Rep. 8, 14728. doi: 10.1038/s41598-018-33016-7, PMID: 30283025 PMC6170476

[ref21] Beirao S. Fernandes S. Coelho J. Faustino M. A. Tome J. P. Neves M. G.. (2014). Photodynamic inactivation of bacterial and yeast biofilms with a cationic porphyrin. Photochem. Photobiol. 90, 1387–1396. doi: 10.1111/php.12331, PMID: 25112506

[ref22] Bilici K. Atac N. Muti A. Baylam I. Dogan O. Sennaroglu A.. (2020). Broad spectrum antibacterial photodynamic and photothermal therapy achieved with indocyanine green loaded SPIONs under near infrared irradiation. Biomater. Sci. 8, 4616–4625. doi: 10.1039/d0bm00821d, PMID: 32676631

[ref23] Bonchi C. Frangipani E. Imperi F. Visca P. (2015). Pyoverdine and proteases affect the response of *Pseudomonas aeruginosa* to gallium in human serum. Antimicrob. Agents Chemother. 59, 5641–5646. doi: 10.1128/AAC.01097-15, PMID: 26149986 PMC4538532

[ref24] Byrd M. S. Sadovskaya I. Vinogradov E. Lu H. Sprinkle A. B. Richardson S. H.. (2009). Genetic and biochemical analyses of the *Pseudomonas aeruginosa* Psl exopolysaccharide reveal overlapping roles for polysaccharide synthesis enzymes in Psl and LPS production. Mol. Microbiol. 73, 622–638. doi: 10.1111/j.1365-2958.2009.06795.x, PMID: 19659934 PMC4409829

[ref25] Chanda W. Joseph T. P. Padhiar A. A. Guo X. Min L. Wang W.. (2017). Combined effect of linolenic acid and tobramycin on *Pseudomonas aeruginosa* biofilm formation and quorum sensing. Exp. Ther. Med. 14, 4328–4338. doi: 10.3892/etm.2017.5110, PMID: 29104645 PMC5658730

[ref26] Chang Y. Wang P. C. Ma H. M. Chen S. Y. Fu Y. H. Liu Y. Y.. (2019). Design, synthesis and evaluation of halogenated furanone derivatives as quorum sensing inhibitors in *Pseudomonas aeruginosa*. Eur. J. Pharm. Sci. 140:105058. doi: 10.1016/j.ejps.2019.105058, PMID: 31472255

[ref27] Chauhan A. Lebeaux D. Ghigo J. M. Beloin C. (2012). Full and broad-spectrum in vivo eradication of catheter-associated biofilms using gentamicin-EDTA antibiotic lock therapy. Antimicrob. Agents Chemother. 56, 6310–6318. doi: 10.1128/AAC.01606-12, PMID: 23027191 PMC3497211

[ref28] Chegini Z. Khoshbayan A. Taati Moghadam M. Farahani I. Jazireian P. Shariati A. (2020). Bacteriophage therapy against *Pseudomonas aeruginosa* biofilms: A review. Ann. Clin. Microbiol. Antimicrob. 19, 45. doi: 10.1186/s12941-020-00389-5, PMID: 32998720 PMC7528332

[ref29] Chou S. Wang J. Shang L. Akhtar M. U. Wang Z. Shi B.. (2019). Short, symmetric-helical peptides have narrow-spectrum activity with low resistance potential and high selectivity. Biomater. Sci. 7, 2394–2409. doi: 10.1039/c9bm00044e, PMID: 30919848

[ref30] Chrzanowski L. Lawniczak L. Czaczyk K. (2012). Why do microorganisms produce rhamnolipids? World J. Microbiol. Biotechnol. 28, 401–419. doi: 10.1007/s11274-011-0854-8, PMID: 22347773 PMC3270259

[ref31] Cieplik F. Deng D. Crielaard W. Buchalla W. Hellwig E. Al-Ahmad A.. (2018). Antimicrobial photodynamic therapy – what we know and what we don't. Crit. Rev. Microbiol. 44, 571–589. doi: 10.1080/1040841X.2018.1467876, PMID: 29749263

[ref32] Clokie M. R. Millard A. D. Letarov A. V. Heaphy S. (2011). Phages in nature. Bacteriophage 1, 31–45. doi: 10.4161/bact.1.1.14942, PMID: 21687533 PMC3109452

[ref33] Colvin K. M. Alnabelseya N. Baker P. Whitney J. C. Howell P. L. Parsek M. R. (2013). PelA deacetylase activity is required for Pel polysaccharide synthesis in *Pseudomonas aeruginosa*. J. Bacteriol. 195, 2329–2339. doi: 10.1128/JB.02150-12, PMID: 23504011 PMC3650530

[ref34] Colvin K. M. Gordon V. D. Murakami K. Borlee B. R. Wozniak D. J. Wong G. C.. (2011). The pel polysaccharide can serve a structural and protective role in the biofilm matrix of *Pseudomonas aeruginosa*. PLoS Pathog. 7:e1001264. doi: 10.1371/journal.ppat.1001264, PMID: 21298031 PMC3029257

[ref35] Colvin K. M. Irie Y. Tart C. S. Urbano R. Whitney J. C. Ryder C.. (2012). The Pel and Psl polysaccharides provide *Pseudomonas aeruginosa* structural redundancy within the biofilm matrix. Environ. Microbiol. 14, 1913–1928. doi: 10.1111/j.1462-2920.2011.02657.x, PMID: 22176658 PMC3840794

[ref36] Costerton J. W. Stewart P. S. Greenberg E. P. (1999). Bacterial biofilms: A common cause of persistent infections. Science 284, 1318–1322. doi: 10.1126/science.284.5418.1318 10334980

[ref37] Cutruzzola F. Frankenberg-Dinkel N. (2016). Origin and impact of nitric oxide in *Pseudomonas aeruginosa* biofilms. J. Bacteriol. 198, 55–65. doi: 10.1128/JB.00371-15, PMID: 26260455 PMC4686190

[ref38] Daboor S. M. Rohde J. R. Cheng Z. (2021). Disruption of the extracellular polymeric network of *Pseudomonas aeruginosa* biofilms by alginate lyase enhances pathogen eradication by antibiotics. J. Cyst. Fibros. 20, 264–270. doi: 10.1016/j.jcf.2020.04.006, PMID: 32482592

[ref39] Das T. Manoharan A. Whiteley G. Glasbey T. Manos J. (2020). “*Pseudomonas aeruginosa* biofilms and infections: roles of extracellular molecules,” in New and Future Developments in Microbial Biotechnology and Bioengineering: Microbial Biofilms. eds. Yadav M. K. Singh B. P. (Amsterdam: Elsevier), 29–46.

[ref40] Das T. Sharma P. K. Busscher H. J. van der Mei H. C. Krom B. P. (2010). Role of extracellular DNA in initial bacterial adhesion and surface aggregation. Appl. Environ. Microbiol. 76, 3405–3408. doi: 10.1128/AEM.03119-09, PMID: 20363802 PMC2869138

[ref41] Davey M. E. Caiazza N. C. O'Toole G. A. (2003). Rhamnolipid surfactant production affects biofilm architecture in *Pseudomonas aeruginosa* PAO1. J. Bacteriol. 185, 1027–1036. doi: 10.1128/JB.185.3.1027-1036.2003, PMID: 12533479 PMC142794

[ref42] de la Fuente-Nunez C. Korolik V. Bains M. Nguyen U. Breidenstein E. B. Horsman S.. (2012). Inhibition of bacterial biofilm formation and swarming motility by a small synthetic cationic peptide. Antimicrob. Agents Chemother. 56, 2696–2704. doi: 10.1128/AAC.00064-12, PMID: 22354291 PMC3346644

[ref43] Di Y. P. Lin Q. Chen C. Montelaro R. C. Doi Y. Deslouches B. (2020). Enhanced therapeutic index of an antimicrobial peptide in mice by increasing safety and activity against multidrug-resistant bacteria. Sci. Adv. 6, eaay6817. doi: 10.1126/sciadv.aay6817, PMID: 32426473 PMC7195177

[ref44] Donlan R. M. (2002). Biofilms: microbial life on surfaces. Emerg. Infect. Dis. 8, 881–890. doi: 10.3201/eid0809.020063, PMID: 12194761 PMC2732559

[ref45] Dosselli R. Millioni R. Puricelli L. Tessari P. Arrigoni G. Franchin C.. (2012). Molecular targets of antimicrobial photodynamic therapy identified by a proteomic approach. J. Proteome 77, 329–343. doi: 10.1016/j.jprot.2012.09.007, PMID: 23000218

[ref46] Drenkard E. (2003). Antimicrobial resistance of *Pseudomonas aeruginosa* biofilms. Microbes Infect. 5, 1213–1219. doi: 10.1016/j.micinf.2003.08.009 14623017

[ref47] Dufour D. Leung V. Lévesque C. (2010). Bacterial biofilm: structure, function, and antimicrobial resistance. Endod. Top. 22, 2–16. doi: 10.1111/j.1601-1546.2012.00277.x

[ref48] Eckhart L. Fischer H. Barken K. B. Tolker-Nielsen T. Tschachler E. (2007). DNase1L2 suppresses biofilm formation by *Pseudomonas aeruginosa* and *Staphylococcus aureus*. Br. J. Dermatol. 156, 1342–1345. doi: 10.1111/j.1365-2133.2007.07886.x, PMID: 17459041

[ref49] Elmanama A. A. Al-Sheboul S. Abu-Dan R. I. (2020). Antimicrobial resistance and biofilm formation of *Pseudomonas aeruginosa*. Int. Arabic J. Antimicrob. Agents 10, 3. doi: 10.3823/846

[ref50] Epstein A. K. Wong T. S. Belisle R. A. Boggs E. M. Aizenberg J. (2012). Liquid-infused structured surfaces with exceptional anti-biofouling performance. Proc. Natl. Acad. Sci. U. S. A. 109, 13182–13187. doi: 10.1073/pnas.1201973109, PMID: 22847405 PMC3421179

[ref51] Feng Y. Coradi Tonon C. Ashraf S. Hasan T. (2021). Photodynamic and antibiotic therapy in combination against bacterial infections: efficacy, determinants, mechanisms, and future perspectives. Adv. Drug Deliv. Rev. 177:113941. doi: 10.1016/j.addr.2021.113941, PMID: 34419503

[ref52] Feng Q. Huang Y. Chen M. Li G. Chen Y. (2015). Functional synergy of alpha-helical antimicrobial peptides and traditional antibiotics against gram-negative and gram-positive bacteria in vitro and in vivo. Eur. J. Clin. Microbiol. Infect. Dis. 34, 197–204. doi: 10.1007/s10096-014-2219-3, PMID: 25169965

[ref53] Ferriol-Gonzalez C. Domingo-Calap P. (2020). Phages for biofilm removal. Antibiotics (Basel) 9, 268. doi: 10.3390/antibiotics9050268, PMID: 32455536 PMC7277876

[ref54] Finkel S. E. Kolter R. (2001). DNA as a nutrient: novel role for bacterial competence gene homologs. J. Bacteriol. 183, 6288–6293. doi: 10.1128/JB.183.21.6288-6293.2001, PMID: 11591672 PMC100116

[ref55] Fleming D. Chahin L. Rumbaugh K. (2017). Glycoside hydrolases degrade polymicrobial bacterial biofilms in wounds. Antimicrob. Agents Chemother. 61, e01998-16. doi: 10.1128/AAC.01998-16, PMID: 27872074 PMC5278739

[ref56] Flemming H. C. Wuertz S. (2019). Bacteria and archaea on earth and their abundance in biofilms. Nat. Rev. Microbiol. 17, 247–260. doi: 10.1038/s41579-019-0158-9, PMID: 30760902

[ref57] Franklin M. J. Nivens D. E. Weadge J. T. Howell P. L. (2011). Biosynthesis of the *Pseudomonas aeruginosa* extracellular polysaccharides, alginate, Pel, and Psl. Front. Microbiol. 2, 167. doi: 10.3389/fmicb.2011.00167, PMID: 21991261 PMC3159412

[ref58] Ghafoor A. Hay I. D. Rehm B. H. (2011). Role of exopolysaccharides in *Pseudomonas aeruginosa* biofilm formation and architecture. Appl. Environ. Microbiol. 77, 5238–5246. doi: 10.1128/AEM.00637-11, PMID: 21666010 PMC3147449

[ref59] Ghorbani H. Memar M. Y. Sefidan F. Y. Yekani M. Ghotaslou R. (2017). In vitro synergy of antibiotic combinations against planktonic and biofilm *Pseudomonas aeruginosa*. GMS Hyg. Infect. Control. 12, Doc17. doi: 10.3205/dgkh000302, PMID: 29094001 PMC5647455

[ref60] Gnanadhas D. P. Elango M. Datey A. Chakravortty D. (2015). Chronic lung infection by *Pseudomonas aeruginosa* biofilm is cured by L-methionine in combination with antibiotic therapy. Sci. Rep. 5, 16043. doi: 10.1038/srep16043, PMID: 26521707 PMC4629202

[ref61] Goss C. H. Kaneko Y. Khuu L. Anderson G. D. Ravishankar S. Aitken M. L.. (2018). Gallium disrupts bacterial iron metabolism and has therapeutic effects in mice and humans with lung infections. Sci. Transl. Med. 10, eaat7520. doi: 10.1126/scitranslmed.aat7520, PMID: 30257953 PMC6637966

[ref62] Harper D. Parracho H. Walker J. Sharp R. Hughes G. Werthén M.. (2014). Bacteriophages and Biofilms. Antibiotics 3, 270–284. doi: 10.3390/antibiotics3030270

[ref63] Hoiby N. Bjarnsholt T. Givskov M. Molin S. Ciofu O. (2010). Antibiotic resistance of bacterial biofilms. Int. J. Antimicrob. Agents 35, 322–332. doi: 10.1016/j.ijantimicag.2009.12.011 20149602

[ref64] Hoiby N. Ciofu O. Johansen H. K. Song Z. J. Moser C. Jensen P. O.. (2011). The clinical impact of bacterial biofilms. Int. J. Oral Sci. 3, 55–65. doi: 10.4248/IJOS11026, PMID: 21485309 PMC3469878

[ref65] Hossain S. Boon E. M. (2017). Discovery of a novel nitric oxide binding protein and nitric-oxide-responsive signaling pathway in *Pseudomonas aeruginosa*. ACS Infect. Dis. 3, 454–461. doi: 10.1021/acsinfecdis.7b00027, PMID: 28238256 PMC5468770

[ref66] Howlin R. P. Cathie K. Hall-Stoodley L. Cornelius V. Duignan C. Allan R. N.. (2017). Low-dose nitric oxide as targeted anti-biofilm adjunctive therapy to treat chronic *Pseudomonas aeruginosa* infection in cystic fibrosis. Mol. Ther. 25, 2104–2116. doi: 10.1016/j.ymthe.2017.06.021, PMID: 28750737 PMC5589160

[ref67] Hu X. Huang Y. Y. Wang Y. Wang X. Hamblin M. R. (2018). Antimicrobial photodynamic therapy to control clinically relevant biofilm infections. Front. Microbiol. 9, 1299. doi: 10.3389/fmicb.2018.01299, PMID: 29997579 PMC6030385

[ref68] Huang H. Shao X. Xie Y. Wang T. Zhang Y. Wang X.. (2019). An integrated genomic regulatory network of virulence-related transcriptional factors in *Pseudomonas aeruginosa*. Nat. Commun. 10, 2931. doi: 10.1038/s41467-019-10778-w, PMID: 31270321 PMC6610081

[ref69] Hughes G. Webber M. A. (2017). Novel approaches to the treatment of bacterial biofilm infections. Br. J. Pharmacol. 174, 2237–2246. doi: 10.1111/bph.13706, PMID: 28063237 PMC5481657

[ref70] Hymes S. R. Randis T. M. Sun T. Y. Ratner A. J. (2013). DNase inhibits *Gardnerella vaginalis* biofilms in vitro and in vivo. J. Infect. Dis. 207, 1491–1497. doi: 10.1093/infdis/jit047, PMID: 23431033 PMC3627197

[ref71] Jamal M. Ahmad W. Andleeb S. Jalil F. Imran M. Nawaz M. A.. (2018). Bacterial biofilm and associated infections. J. Chin. Med. Assoc. 81, 7–11. doi: 10.1016/j.jcma.2017.07.012 29042186

[ref72] Jennings L. K. Storek K. M. Ledvina H. E. Coulon C. Marmont L. S. Sadovskaya I.. (2015). Pel is a cationic exopolysaccharide that cross-links extracellular DNA in the *Pseudomonas aeruginosa* biofilm matrix. Proc. Natl. Acad. Sci. U. S. A. 112, 11353–11358. doi: 10.1073/pnas.1503058112, PMID: 26311845 PMC4568648

[ref73] Jiang Y. Geng M. Bai L. (2020). Targeting biofilms therapy: current research strategies and development hurdles. Microorganisms 8, 1222. doi: 10.3390/microorganisms8081222, PMID: 32796745 PMC7465149

[ref74] Jiang P. Li J. Han F. Duan G. Lu X. Gu Y.. (2011). Antibiofilm activity of an exopolysaccharide from marine bacterium vibrio sp. QY101. PLoS One 6:e18514. doi: 10.1371/journal.pone.0018514, PMID: 21490923 PMC3072402

[ref75] Kalia V. C. (2013). Quorum sensing inhibitors: An overview. Biotechnol. Adv. 31, 224–245. doi: 10.1016/j.biotechadv.2012.10.004 23142623

[ref76] Kamal A. A. M. Maurer C. K. Allegretta G. Haupenthal J. Empting M. Hartmann R. W. (2017). “Quorum sensing inhibitors as pathoblockers for *Pseudomonas aeruginosa* infections: A new concept in anti-infective drug discovery,” in Antibacterials, Topics in Medicinal Chemistry. eds. Fisher J. Mobashery S. Miller M. (Cham: Springer), 185–210.

[ref77] Kamaruzzaman N. F. Tan L. P. Mat Yazid K. A. Saeed S. I. Hamdan R. H. Choong S. S.. (2018). Targeting the bacterial protective armour; challenges and novel strategies in the treatment of microbial biofilm. Materials (Basel) 11, 1705. doi: 10.3390/ma11091705, PMID: 30217006 PMC6164881

[ref78] Kaneko Y. Thoendel M. Olakanmi O. Britigan B. E. Singh P. K. (2007). The transition metal gallium disrupts *Pseudomonas aeruginosa* iron metabolism and has antimicrobial and antibiofilm activity. J. Clin. Invest. 117, 877–888. doi: 10.1172/JCI30783, PMID: 17364024 PMC1810576

[ref79] Kang D. Revtovich A. V. Deyanov A. E. Kirienko N. V. (2021). Pyoverdine inhibitors and gallium nitrate synergistically affect *Pseudomonas aeruginosa*. mSphere 6:e0040121. doi: 10.1128/mSphere.00401-21, PMID: 34133200 PMC8265654

[ref80] Khan F. Pham D. T. N. Kim Y. M. (2020). Alternative strategies for the application of aminoglycoside antibiotics against the biofilm-forming human pathogenic bacteria. Appl. Microbiol. Biotechnol. 104, 1955–1976. doi: 10.1007/s00253-020-10360-1, PMID: 31970432

[ref81] Kim S. K. Lee J. H. (2016). Biofilm dispersion in *Pseudomonas aeruginosa*. J. Microbiol. 54, 71–85. doi: 10.1007/s12275-016-5528-7 26832663

[ref82] Kitao T. Lepine F. Babloudi S. Walte F. Steinbacher S. Maskos K.. (2018). Molecular insights into function and competitive inhibition of *Pseudomonas aeruginosa* multiple virulence factor regulator. mBio 9, e02158-17. doi: 10.1128/mBio.02158-17, PMID: 29339431 PMC5770554

[ref83] Koo H. Allan R. N. Howlin R. P. Stoodley P. Hall-Stoodley L. (2017). Targeting microbial biofilms: current and prospective therapeutic strategies. Nat. Rev. Microbiol. 15, 740–755. doi: 10.1038/nrmicro.2017.99, PMID: 28944770 PMC5685531

[ref84] Kostakioti M. Hadjifrangiskou M. Hultgren S. J. (2013). Bacterial biofilms: development, dispersal, and therapeutic strategies in the dawn of the postantibiotic era. Cold Spring Harb. Perspect. Med. 3:a010306. doi: 10.1101/cshperspect.a010306, PMID: 23545571 PMC3683961

[ref85] Kovach K. N. Fleming D. Wells M. J. Rumbaugh K. P. Gordon V. D. (2020). Specific disruption of established *Pseudomonas aeruginosa* biofilms using polymer-attacking enzymes. Langmuir 36, 1585–1595. doi: 10.1021/acs.langmuir.9b02188, PMID: 31990563 PMC7063831

[ref86] Kumar A. Alam A. Rani M. Ehtesham N. Z. Hasnain S. E. (2017). Biofilms: survival and defense strategy for pathogens. Int. J. Med. Microbiol. 307, 481–489. doi: 10.1016/j.ijmm.2017.09.016, PMID: 28950999

[ref87] Kumar L. Chhibber S. Harjai K. (2013). Zingerone inhibit biofilm formation and improve antibiofilm efficacy of ciprofloxacin against *Pseudomonas aeruginosa* PAO1. Fitoterapia 90, 73–78. doi: 10.1016/j.fitote.2013.06.017, PMID: 23831483

[ref88] Kumar L. Chhibber S. Kumar R. Kumar M. Harjai K. (2015). Zingerone silences quorum sensing and attenuates virulence of *Pseudomonas aeruginosa*. Fitoterapia 102, 84–95. doi: 10.1016/j.fitote.2015.02.002, PMID: 25704369

[ref89] Kutateladze M. Adamia R. (2010). Bacteriophages as potential new therapeutics to replace or supplement antibiotics. Trends Biotechnol. 28, 591–595. doi: 10.1016/j.tibtech.2010.08.001 20810181

[ref90] Kutter E. De Vos D. Gvasalia G. Alavidze Z. Gogokhia L. Kuhl S.. (2010). Phage therapy in clinical practice: treatment of human infections. Curr. Pharm. Biotechnol. 11, 69–86. doi: 10.2174/138920110790725401 20214609

[ref91] Lashua L. P. Melvin J. A. Deslouches B. Pilewski J. M. Montelaro R. C. Bomberger J. M. (2016). Engineered cationic antimicrobial peptide (eCAP) prevents *Pseudomonas aeruginosa* biofilm growth on airway epithelial cells. J. Antimicrob. Chemother. 71, 2200–2207. doi: 10.1093/jac/dkw143, PMID: 27231279 PMC4954927

[ref92] Le C. F. Fang C. M. Sekaran S. D. (2017). Intracellular targeting mechanisms by antimicrobial peptides. Antimicrob. Agents Chemother. 61, e02340-16. doi: 10.1128/AAC.02340-16, PMID: 28167546 PMC5365711

[ref93] Lebeaux D. Leflon-Guibout V. Ghigo J. M. Beloin C. (2015). In vitro activity of gentamicin, vancomycin or amikacin combined with EDTA or l-arginine as lock therapy against a wide spectrum of biofilm-forming clinical strains isolated from catheter-related infections. J. Antimicrob. Chemother. 70, 1704–1712. doi: 10.1093/jac/dkv044, PMID: 25712314

[ref94] Lee K. Yoon S. S. (2017). *Pseudomonas aeruginosa* biofilm, a programmed bacterial life for fitness. J. Microbiol. Biotechnol. 27, 1053–1064. doi: 10.4014/jmb.1611.11056, PMID: 28301918

[ref95] Lee J. Zhang L. (2015). The hierarchy quorum sensing network in *Pseudomonas aeruginosa*. Protein Cell 6, 26–41. doi: 10.1007/s13238-014-0100-x, PMID: 25249263 PMC4286720

[ref96] Lequette Y. Greenberg E. P. (2005). Timing and localization of rhamnolipid synthesis gene expression in *Pseudomonas aeruginosa* biofilms. J. Bacteriol. 187, 37–44. doi: 10.1128/JB.187.1.37-44.2005, PMID: 15601686 PMC538809

[ref97] Li W. Geng X. Liu D. Li Z. (2019). Near-infrared light-enhanced protease-conjugated gold nanorods as a photothermal antimicrobial agent for elimination of exotoxin and biofilms. Int. J. Nanomedicine 14, 8047–8058. doi: 10.2147/IJN.S212750, PMID: 31632017 PMC6781946

[ref98] Li Y. Xiao P. Wang Y. Hao Y. (2020). Mechanisms and control measures of mature biofilm resistance to antimicrobial agents in the clinical context. ACS Omega 5, 22684–22690. doi: 10.1021/acsomega.0c02294, PMID: 32954115 PMC7495453

[ref99] Liang X. Zou Z. Zou Z. Li C. Dong X. Yin H.. (2020). Effect of antibacterial photodynamic therapy on *Streptococcus mutans* plaque biofilm *in vitro*. J. Innov. Opt. Health Sci. 13, 2050022. doi: 10.1142/S1793545820500224

[ref100] Lichtenberg M. Jakobsen T. H. Kuhl M. Kolpen M. Jensen P. O. Bjarnsholt T. (2022). The structure-function relationship of *Pseudomonas aeruginosa* in infections and its influence on the microenvironment. FEMS Microbiol. Rev. doi: 10.1093/femsre/fuac018 [Epub ahead of print], PMID: 35472245 PMC9438473

[ref101] Limoli D. H. Jones C. J. Wozniak D. J. (2015). Bacterial extracellular polysaccharides in biofilm formation and function. Microbiol. Spectr. 3, MB-0011–2014. doi: 10.1128/microbiolspec.MB-0011-2014, PMID: 26185074 PMC4657554

[ref102] Limoli D. H. Rockel A. B. Host K. M. Jha A. Kopp B. T. Hollis T.. (2014). Cationic antimicrobial peptides promote microbial mutagenesis and pathoadaptation in chronic infections. PLoS Pathog. 10:e1004083. doi: 10.1371/journal.ppat.1004083, PMID: 24763694 PMC3999168

[ref103] Lin J. Cheng J. (2019). “Quorum sensing in *Pseudomonas aeruginosa* and its relationship to biofilm development,” in Introduction to Biofilm Engineering. eds. Rathinam N. K. Sani R. K. (Washington, DC: ACS Publications), 1–16.

[ref104] Lin J. Cheng J. Wang Y. Shen X. (2018a). The *pseudomonas* quinolone signal (PQS): not just for quorum sensing anymore. Front. Cell. Infect. Microbiol. 8, 230. doi: 10.3389/fcimb.2018.00230, PMID: 30023354 PMC6039570

[ref105] Lin Q. Deslouches B. Montelaro R. C. Di Y. P. (2018b). Prevention of ESKAPE pathogen biofilm formation by antimicrobial peptides WLBU2 and LL37. Int. J. Antimicrob. Agents 52, 667–672. doi: 10.1016/j.ijantimicag.2018.04.019, PMID: 29753132 PMC6230315

[ref106] Lin Q. Pilewski J. M. Di Y. P. (2021). Acidic microenvironment determines antibiotic susceptibility and biofilm formation of *Pseudomonas aeruginosa*. Front. Microbiol. 12:747834. doi: 10.3389/fmicb.2021.747834, PMID: 34867864 PMC8640179

[ref107] Llamas M. A. Sparrius M. Kloet R. Jimenez C. R. Vandenbroucke-Grauls C. Bitter W. (2006). The heterologous siderophores ferrioxamine B and ferrichrome activate signaling pathways in *Pseudomonas aeruginosa*. J. Bacteriol. 188, 1882–1891. doi: 10.1128/JB.188.5.1882-1891.2006, PMID: 16484199 PMC1426570

[ref108] Ma L. Conover M. Lu H. Parsek M. R. Bayles K. Wozniak D. J. (2009). Assembly and development of the *Pseudomonas aeruginosa* biofilm matrix. PLoS Pathog. 5:e1000354. doi: 10.1371/journal.ppat.1000354, PMID: 19325879 PMC2654510

[ref109] Ma L. Jackson K. D. Landry R. M. Parsek M. R. Wozniak D. J. (2006). Analysis of *Pseudomonas aeruginosa* conditional Psl variants reveals roles for the Psl polysaccharide in adhesion and maintaining biofilm structure postattachment. J. Bacteriol. 188, 8213–8221. doi: 10.1128/JB.01202-06, PMID: 16980452 PMC1698210

[ref110] Ma L. Z. Wang D. Liu Y. Zhang Z. Wozniak D. J. (2022). Regulation of biofilm exopolysaccharide biosynthesis and degradation in *Pseudomonas aeruginosa*. Annu. Rev. Microbiol. 76, 413–433. doi: 10.1146/annurev-micro-041320-111355 35655342

[ref111] Ma L. Wang S. Wang D. Parsek M. R. Wozniak D. J. (2012). The roles of biofilm matrix polysaccharide Psl in mucoid *Pseudomonas aeruginosa* biofilms. FEMS Immunol. Med. Microbiol. 65, 377–380. doi: 10.1111/j.1574-695X.2012.00934.x, PMID: 22309106

[ref112] Mahlapuu M. Hakansson J. Ringstad L. Bjorn C. (2016). Antimicrobial peptides: An emerging category of therapeutic agents. Front. Cell. Infect. Microbiol. 6, 194. doi: 10.3389/fcimb.2016.00194, PMID: 28083516 PMC5186781

[ref113] Mann E. E. Wozniak D. J. (2012). *Pseudomonas* biofilm matrix composition and niche biology. FEMS Microbiol. Rev. 36, 893–916. doi: 10.1111/j.1574-6976.2011.00322.x, PMID: 22212072 PMC4409827

[ref114] Marmont L. S. Whitfield G. B. Rich J. D. Yip P. Giesbrecht L. B. Stremick C. A.. (2017). PelA and PelB proteins form a modification and secretion complex essential for Pel polysaccharide-dependent biofilm formation in *Pseudomonas aeruginosa*. J. Biol. Chem. 292, 19411–19422. doi: 10.1074/jbc.M117.812842, PMID: 28972168 PMC5702679

[ref115] Marshall B. Stintzi A. Gilmour C. Meyer J. M. Poole K. (2009). Citrate-mediated iron uptake in *Pseudomonas aeruginosa*: involvement of the citrate-inducible FecA receptor and the FeoB ferrous iron transporter. Microbiology (Reading) 155, 305–315. doi: 10.1099/mic.0.023531-0, PMID: 19118371

[ref116] Martinez M. Goncalves S. Felicio M. R. Maturana P. Santos N. C. Semorile L.. (2019). Synergistic and antibiofilm activity of the antimicrobial peptide P5 against carbapenem-resistant *Pseudomonas aeruginosa*. Biochim. Biophys. Acta Biomembr. 1861, 1329–1337. doi: 10.1016/j.bbamem.2019.05.008, PMID: 31095945

[ref117] Maura D. Rahme L. G. (2017). Pharmacological inhibition of the *Pseudomonas aeruginosa* MvfR quorum-sensing system interferes with biofilm formation and potentiates antibiotic-mediated biofilm disruption. Antimicrob. Agents Chemother. 61, e01362-17. doi: 10.1128/AAC.01362-17, PMID: 28923875 PMC5700327

[ref118] Maurice N. M. Bedi B. Sadikot R. T. (2018). *Pseudomonas aeruginosa* biofilms: Host response and clinical implications in lung infections. Am. J. Respir. Cell Mol. Biol. 58, 428–439. doi: 10.1165/rcmb.2017-0321TR, PMID: 29372812 PMC5894500

[ref119] McDougald D. Rice S. A. Barraud N. Steinberg P. D. Kjelleberg S. (2011). Should we stay or should we go: mechanisms and ecological consequences for biofilm dispersal. Nat. Rev. Microbiol. 10, 39–50. doi: 10.1038/nrmicro2695 22120588

[ref120] Melo L. D. R. Pires D. P. Monteiro R. Azeredo J. (2019). “Phage therapy of infectious biofilms: challenges and strategies,” in Phage Therapy: A Practical Approach. eds. Górski A. Międzybrodzki R. Borysowski J. (Cham: Springer), 295–313.

[ref121] Mi L. Liu Y. Wang C. He T. Gao S. Xing S.. (2019). Identification of a lytic *Pseudomonas aeruginosa* phage depolymerase and its anti-biofilm effect and bactericidal contribution to serum. Virus Genes 55, 394–405. doi: 10.1007/s11262-019-01660-4, PMID: 30937696

[ref122] Minandri F. Bonchi C. Frangipani E. Imperi F. Visca P. (2014). Promises and failures of gallium as an antibacterial agent. Future Microbiol. 9, 379–397. doi: 10.2217/fmb.14.3, PMID: 24762310

[ref123] Mitchell K. F. Zarnowski R. Andes D. R. (2016). Fungal super glue: the biofilm matrix and its composition, assembly, and functions. PLoS Pathog. 12:e1005828. doi: 10.1371/journal.ppat.1005828, PMID: 27685086 PMC5042517

[ref124] Moradali M. F. Ghods S. Rehm B. H. (2017). *Pseudomonas aeruginosa* lifestyle: A paradigm for adaptation, survival, and persistence. Front. Cell. Infect. Microbiol. 7, 39. doi: 10.3389/fcimb.2017.00039, PMID: 28261568 PMC5310132

[ref125] Moradali M. F. Rehm B. H. (2019). “The role of alginate in bacterial biofilm formation,” in Extracellular Sugar-Based Biopolymers Matrices, Biologically-Inspired Systems. eds. Cohen E. Merzendorfer H. (Cham: Springer), 517–537.

[ref126] Muhlen S. Dersch P. (2016). Anti-virulence strategies to target bacterial infections. Curr. Top. Microbiol. Immunol. 398, 147–183. doi: 10.1007/82_2015_490 26942418

[ref127] O'Brien K. T. Noto J. G. Nichols-O'Neill L. Perez L. J. (2015). Potent irreversible inhibitors of LasR quorum sensing in *Pseudomonas aeruginosa*. ACS Med. Chem. Lett. 6, 162–167. doi: 10.1021/ml500459f, PMID: 25699144 PMC4329587

[ref128] Okshevsky M. Regina V. R. Meyer R. L. (2015). Extracellular DNA as a target for biofilm control. Curr. Opin. Biotechnol. 33, 73–80. doi: 10.1016/j.copbio.2014.12.002, PMID: 25528382

[ref129] Olivares E. Badel-Berchoux S. Provot C. Prevost G. Bernardi T. Jehl F. (2019). Clinical impact of antibiotics for the treatment of *Pseudomonas aeruginosa* biofilm infections. Front. Microbiol. 10, 2894. doi: 10.3389/fmicb.2019.02894, PMID: 31998248 PMC6962142

[ref130] Overhage J. Campisano A. Bains M. Torfs E. C. Rehm B. H. Hancock R. E. (2008). Human host defense peptide LL-37 prevents bacterial biofilm formation. Infect. Immun. 76, 4176–4182. doi: 10.1128/IAI.00318-08, PMID: 18591225 PMC2519444

[ref131] Pang Z. Raudonis R. Glick B. R. Lin T. J. Cheng Z. (2019). Antibiotic resistance in *Pseudomonas aeruginosa*: mechanisms and alternative therapeutic strategies. Biotechnol. Adv. 37, 177–192. doi: 10.1016/j.biotechadv.2018.11.013, PMID: 30500353

[ref132] Pang Z. Zhu Q. (2021). Traditional Chinese medicine is an alternative therapeutic option for treatment of *Pseudomonas aeruginosa* infections. Front. Pharmacol. 12:737252. doi: 10.3389/fphar.2021.737252, PMID: 34512364 PMC8429605

[ref133] Pei R. Lamas-Samanamud G. R. (2014). Inhibition of biofilm formation by T7 bacteriophages producing quorum-quenching enzymes. Appl. Environ. Microbiol. 80, 5340–5348. doi: 10.1128/AEM.01434-14, PMID: 24951790 PMC4136088

[ref134] Perez-Laguna V. Garcia-Luque I. Ballesta S. Perez-Artiaga L. Lampaya-Perez V. Rezusta A.. (2020). Photodynamic therapy using methylene blue, combined or not with gentamicin, against *Staphylococcus aureus* and *Pseudomonas aeruginosa*. Photodiagn. Photodyn. Ther. 31:101810. doi: 10.1016/j.pdpdt.2020.101810 32437976

[ref135] Pires D. P. Oliveira H. Melo L. D. Sillankorva S. Azeredo J. (2016). Bacteriophage-encoded depolymerases: their diversity and biotechnological applications. Appl. Microbiol. Biotechnol. 100, 2141–2151. doi: 10.1007/s00253-015-7247-0, PMID: 26767986

[ref136] Poh W. H. Rice S. A. (2022). Recent developments in nitric oxide donors and delivery for antimicrobial and anti-biofilm applications. Molecules 27, 674. doi: 10.3390/molecules27030674, PMID: 35163933 PMC8839391

[ref137] Pontes J. T. C. Toledo Borges A. B. Roque-Borda C. A. Pavan F. R. (2022). Antimicrobial peptides as an alternative for the eradication of bacterial biofilms of multi-drug resistant bacteria. Pharmaceutics 14, 642. doi: 10.3390/pharmaceutics14030642, PMID: 35336016 PMC8950055

[ref138] Qi L. Christopher G. F. (2019). Role of flagella, type IV Pili, biosurfactants, and extracellular polymeric substance polysaccharides on the formation of pellicles by *Pseudomonas aeruginosa*. Langmuir 35, 5294–5304. doi: 10.1021/acs.langmuir.9b00271, PMID: 30883129

[ref139] Qin Z. Yang L. Qu D. Molin S. Tolker-Nielsen T. (2009). *Pseudomonas aeruginosa* extracellular products inhibit staphylococcal growth, and disrupt established biofilms produced by *Staphylococcus* epidermidis. Microbiology (Reading) 155, 2148–2156. doi: 10.1099/mic.0.028001-0, PMID: 19389780

[ref140] Rahmani-Badi A. Sepehr S. Mohammadi P. Soudi M. R. Babaie-Naiej H. Fallahi H. (2014). A combination of cis-2-decenoic acid and antibiotics eradicates pre-established catheter-associated biofilms. J. Med. Microbiol. 63, 1509–1516. doi: 10.1099/jmm.0.075374-0, PMID: 25082943

[ref141] Rajesh S. Koshi E. Philip K. Mohan A. (2011). Antimicrobial photodynamic therapy: An overview. J. Indian Soc. Periodontol. 15, 323–327. doi: 10.4103/0972-124X.92563, PMID: 22368354 PMC3283927

[ref142] Rajkumari J. Borkotoky S. Murali A. Suchiang K. Mohanty S. K. Busi S. (2018). Cinnamic acid attenuates quorum sensing associated virulence factors and biofilm formation in *Pseudomonas aeruginosa* PAO1. Biotechnol. Lett. 40, 1087–1100. doi: 10.1007/s10529-018-2557-9, PMID: 29680931

[ref143] Redman W. K. Welch G. S. Rumbaugh K. P. (2020). Differential efficacy of glycoside hydrolases to disperse biofilms. Front. Cell. Infect. Microbiol. 10, 379. doi: 10.3389/fcimb.2020.00379, PMID: 32793516 PMC7393775

[ref144] Rendueles O. Kaplan J. B. Ghigo J. M. (2013). Antibiofilm polysaccharides. Environ. Microbiol. 15, 334–346. doi: 10.1111/j.1462-2920.2012.02810.x, PMID: 22730907 PMC3502681

[ref145] Roy R. Tiwari M. Donelli G. Tiwari V. (2018). Strategies for combating bacterial biofilms: A focus on anti-biofilm agents and their mechanisms of action. Virulence 9, 522–554. doi: 10.1080/21505594.2017.1313372, PMID: 28362216 PMC5955472

[ref146] Rumbaugh K. P. Sauer K. (2020). Biofilm dispersion. Nat. Rev. Microbiol. 18, 571–586. doi: 10.1038/s41579-020-0385-0, PMID: 32533131 PMC8564779

[ref147] Rutherford S. T. Bassler B. L. (2012). Bacterial quorum sensing: its role in virulence and possibilities for its control. Cold Spring Harb. Perspect. Med. 2:a012427. doi: 10.1101/cshperspect.a012427, PMID: 23125205 PMC3543102

[ref148] Rzhepishevska O. Ekstrand-Hammarstrom B. Popp M. Bjorn E. Bucht A. Sjostedt A.. (2011). The antibacterial activity of Ga^3+^ is influenced by ligand complexation as well as the bacterial carbon source. Antimicrob. Agents Chemother. 55, 5568–5580. doi: 10.1128/AAC.00386-11, PMID: 21947396 PMC3232821

[ref149] Saxena P. Joshi Y. Rawat K. Bisht R. (2019). Biofilms: Architecture, resistance, quorum sensing and control mechanisms. Indian J. Microbiol. 59, 3–12. doi: 10.1007/s12088-018-0757-6, PMID: 30728625 PMC6328408

[ref150] Schmelcher M. Donovan D. M. Loessner M. J. (2012). Bacteriophage endolysins as novel antimicrobials. Future Microbiol. 7, 1147–1171. doi: 10.2217/fmb.12.97, PMID: 23030422 PMC3563964

[ref151] Schutz C. Empting M. (2018). Targeting the *pseudomonas* quinolone signal quorum sensing system for the discovery of novel anti-infective pathoblockers. Beilstein J. Org. Chem. 14, 2627–2645. doi: 10.3762/bjoc.14.241, PMID: 30410625 PMC6204780

[ref152] Schutz C. Ho D. K. Hamed M. M. Abdelsamie A. S. Rohrig T. Herr C.. (2021). A new PqsR inverse agonist potentiates tobramycin efficacy to eradicate *Pseudomonas aeruginosa* biofilms. Adv. Sci. (Weinh) 8:e2004369. doi: 10.1002/advs.202004369, PMID: 34165899 PMC8224453

[ref153] Seyfi R. Kahaki F. A. Ebrahimi T. Montazersaheb S. Eyvazi S. Babaeipour V.. (2020). Antimicrobial peptides (AMPs): roles, functions and mechanism of action. Int. J. Pept. Res. Ther. 26, 1451–1463. doi: 10.1007/s10989-019-09946-9

[ref154] Shao X. Xie Y. Zhang Y. Liu J. Ding Y. Wu M.. (2020). Novel therapeutic strategies for treating *Pseudomonas aeruginosa* infection. Expert Opin. Drug Discov. 15, 1403–1423. doi: 10.1080/17460441.2020.1803274, PMID: 32880507

[ref155] Sharma D. Misba L. Khan A. U. (2019). Antibiotics versus biofilm: An emerging battleground in microbial communities. Antimicrob. Resist. Infect. Control 8, 76. doi: 10.1186/s13756-019-0533-3, PMID: 31131107 PMC6524306

[ref156] Sharma K. Pagedar Singh A. (2018). Antibiofilm effect of DNase against single and mixed species biofilm. Foods 7, 42. doi: 10.3390/foods7030042, PMID: 29562719 PMC5867557

[ref157] Shrestha L. Fan H. M. Tao H. R. Huang J. D. (2022). Recent strategies to combat biofilms using antimicrobial agents and therapeutic approaches. Pathogens 11, 292. doi: 10.3390/pathogens11030292, PMID: 35335616 PMC8955104

[ref158] Shrivastava S. Shrivastava P. S. Ramasamy J. (2018). World health organization releases global priority list of antibiotic-resistant bacteria to guide research, discovery, and development of new antibiotics. J. Med. Soc. 32, 76. doi: 10.4103/jms.jms_25_17

[ref159] Smith W. D. Bardin E. Cameron L. Edmondson C. L. Farrant K. V. Martin I.. (2017). Current and future therapies for *Pseudomonas aeruginosa* infection in patients with cystic fibrosis. FEMS Microbiol. Lett. 364, fnx121. doi: 10.1093/femsle/fnx121, PMID: 28854668

[ref160] Soothill J. (2013). Use of bacteriophages in the treatment of *Pseudomonas aeruginosa* infections. Expert Rev. Anti Infect. Ther. 11, 909–915. doi: 10.1586/14787210.2013.826990, PMID: 24053272

[ref161] Soren O. Rineh A. Silva D. G. Cai Y. Howlin R. P. Allan R. N.. (2020). Cephalosporin nitric oxide-donor prodrug DEA-C3D disperses biofilms formed by clinical cystic fibrosis isolates of *Pseudomonas aeruginosa*. J. Antimicrob. Chemother. 75, 117–125. doi: 10.1093/jac/dkz378, PMID: 31682251 PMC6910178

[ref162] Soukarieh F. Williams P. Stocks M. J. Camara M. (2018). *Pseudomonas aeruginosa* quorum sensing systems as drug discovery targets: current position and future perspectives. J. Med. Chem. 61, 10385–10402. doi: 10.1021/acs.jmedchem.8b00540, PMID: 29999316

[ref163] Srinivasan R. Santhakumari S. Poonguzhali P. Geetha M. Dyavaiah M. Xiangmin L. (2021). Bacterial biofilm inhibition: A focused review on recent therapeutic strategies for combating the biofilm mediated infections. Front. Microbiol. 12:676458. doi: 10.3389/fmicb.2021.676458, PMID: 34054785 PMC8149761

[ref164] Swartjes J. J. Das T. Sharifi S. Subbiahdoss G. Sharma P. K. Krom B. P.. (2013). A functional DNase I coating to prevent adhesion of bacteria and the formation of biofilm. Adv. Funct. Mater. 23, 2843–2849. doi: 10.1002/adfm.201202927

[ref165] Tahmassebi J. F. Drogkari E. Wood S. R. (2015). A study of the control of oral plaque biofilms via antibacterial photodynamic therapy. Eur. Arch. Paediatr. Dent. 16, 433–440. doi: 10.1007/s40368-014-0165-5, PMID: 26385341

[ref166] Talapko J. Skrlec I. (2020). The principles, mechanisms, and benefits of unconventional agents in the treatment of biofilm infection. Pharmaceuticals (Basel) 13, 299. doi: 10.3390/ph13100299, PMID: 33050521 PMC7600518

[ref167] Thi M. T. T. Wibowo D. Rehm B. H. A. (2020). *Pseudomonas aeruginosa* biofilms. Int. J. Mol. Sci. 21, 8671. doi: 10.3390/ijms21228671, PMID: 33212950 PMC7698413

[ref168] Tovar-Garcia A. Angarita-Zapata V. Cazares A. Jasso-Chavez R. Belmont-Diaz J. Sanchez-Torres V.. (2020). Characterization of gallium resistance induced in a *Pseudomonas aeruginosa* cystic fibrosis isolate. Arch. Microbiol. 202, 617–622. doi: 10.1007/s00203-019-01777-y, PMID: 31773196

[ref169] Tummler B. (2019). Emerging therapies against infections with *Pseudomonas aeruginosa*. F1000Res 8:1371. doi: 10.12688/f1000research.19509.1, PMID: 31448090 PMC6688719

[ref170] Tuon F. F. Dantas L. R. Suss P. H. Tasca Ribeiro V. S. (2022). Pathogenesis of the *Pseudomonas aeruginosa* biofilm: A review. Pathogens 11, 300. doi: 10.3390/pathogens11030300, PMID: 35335624 PMC8950561

[ref171] Vuotto C. Donelli G. (2019). Novel treatment strategies for biofilm-based infections. Drugs 79, 1635–1655. doi: 10.1007/s40265-019-01184-z, PMID: 31468316

[ref172] Wagner S. Sommer R. Hinsberger S. Lu C. Hartmann R. W. Empting M.. (2016). Novel strategies for the treatment of *Pseudomonas aeruginosa* infections. J. Med. Chem. 59, 5929–5969. doi: 10.1021/acs.jmedchem.5b01698, PMID: 26804741

[ref173] Wainwright M. Maisch T. Nonell S. Plaetzer K. Almeida A. Tegos G. P.. (2017). Photoantimicrobials-are we afraid of the light? Lancet Infect. Dis. 17, e49–e55. doi: 10.1016/s1473-3099(16)30268-7, PMID: 27884621 PMC5280084

[ref174] Wang L. Di Luca M. Tkhilaishvili T. Trampuz A. Gonzalez Moreno M. (2019). Synergistic activity of Fosfomycin, ciprofloxacin, and gentamicin Against *Escherichia coli* and *Pseudomonas aeruginosa* biofilms. Front. Microbiol. 10, 2522. doi: 10.3389/fmicb.2019.02522, PMID: 31781056 PMC6853019

[ref175] Wang L. Fan X. Gonzalez Moreno M. Tkhilaishvili T. Du W. Zhang X.. (2022). Photocatalytic quantum dot-armed bacteriophage for combating drug-resistant bacterial infection. Adv. Sci. (Weinh) 9:e2105668. doi: 10.1002/advs.202105668, PMID: 35434949 PMC9189633

[ref176] Wang S. Gao Q. Cheng J. Lin J. (2020). Regulation of *Pseudomonas aeruginosa* biofilms by quorum sensing systems and c-di-GMP. Acta Microbiol. Sin. 61, 1106–1122. doi: 10.13343/j.cnki.wsxb.20200367

[ref177] Wang S. Liu X. Liu H. Zhang L. Guo Y. Yu S.. (2015). The exopolysaccharide Psl-eDNA interaction enables the formation of a biofilm skeleton in *Pseudomonas aeruginosa*. Environ. Microbiol. Rep. 7, 330–340. doi: 10.1111/1758-2229.12252, PMID: 25472701 PMC4656019

[ref178] Wang S. Niu Y. Zhang H. Li P. Zhang N. Cheng J.. (2021). An engineered bacterium for the targeted delivery of proteins to destroy *Pseudomonas aeruginosa* biofilms. Acta Microbiol. Sin. 61, 2726–2748. doi: 10.13343/j.cnki.wsxb.20200680

[ref179] Wei Q. Ma L. Z. (2013). Biofilm matrix and its regulation in *Pseudomonas aeruginosa*. Int. J. Mol. Sci. 14, 20983–21005. doi: 10.3390/ijms141020983, PMID: 24145749 PMC3821654

[ref180] Wen Y. L. Wu B. J. Kao P. H. Fu Y. S. Chang L. S. (2013). Antibacterial and membrane-damaging activities of beta-bungarotoxin B chain. J. Pept. Sci. 19, 1–8. doi: 10.1002/psc.2463, PMID: 23136049

[ref181] Whitchurch C. B. Tolker-Nielsen T. Ragas P. C. Mattick J. S. (2002). Extracellular DNA required for bacterial biofilm formation. Science 295, 1487. doi: 10.1126/science.295.5559.1487 11859186

[ref182] Whiteley M. Diggle S. P. Greenberg E. P. (2017). Progress in and promise of bacterial quorum sensing research. Nature 551, 313–320. doi: 10.1038/nature24624, PMID: 29144467 PMC5870893

[ref183] Wi Y. M. Patel R. (2018). Understanding biofilms and novel approaches to the diagnosis, prevention, and treatment of medical device-associated infections. Infect. Dis. Clin. N. Am. 32, 915–929. doi: 10.1016/j.idc.2018.06.009, PMID: 30241715 PMC6215726

[ref184] Williams D. E. Boon E. M. (2019). Towards understanding the molecular basis of nitric oxide-regulated group behaviors in pathogenic bacteria. J. Innate Immun. 11, 205–215. doi: 10.1159/000494740, PMID: 30557874 PMC6487220

[ref185] Wilton M. Charron-Mazenod L. Moore R. Lewenza S. (2016). Extracellular DNA acidifies biofilms and induces aminoglycoside resistance in *Pseudomonas aeruginosa*. Antimicrob. Agents Chemother. 60, 544–553. doi: 10.1128/AAC.01650-15, PMID: 26552982 PMC4704225

[ref186] Wu H. Moser C. Wang H. Z. Hoiby N. Song Z. J. (2015). Strategies for combating bacterial biofilm infections. Int. J. Oral Sci. 7, 1–7. doi: 10.1038/ijos.2014.65, PMID: 25504208 PMC4817533

[ref187] Wu Z. Zheng R. Zhang J. Wu S. (2021). Transcriptional profiling of *Pseudomonas aeruginosa* PAO1 in response to anti-biofilm and anti-infection agent exopolysaccharide EPS273. J. Appl. Microbiol. 130, 265–277. doi: 10.1111/jam.14764, PMID: 32619289

[ref188] Xiu W. Shan J. Yang K. Xiao H. Yu W. Li H.. (2021). Recent development of nanomedicine for the treatment of bacterial biofilm infections. Viewpoints 2, 20200065. doi: 10.1002/VIW.20200065

[ref189] Yadav J. Kumari R. M. Verma V. Nimesh S. (2021). Recent development in therapeutic strategies targeting *Pseudomonas aeruginosa* biofilms – a review. Mater. Today Proc. 46, 2359–2373. doi: 10.1016/j.matpr.2021.05.245

[ref190] Yan J. Mao J. Xie J. (2014). Bacteriophage polysaccharide depolymerases and biomedical applications. BioDrugs 28, 265–274. doi: 10.1007/s40259-013-0081-y, PMID: 24352884

[ref191] Yang Y. Qi P. K. Yang Z. L. Huang N. (2015). Nitric oxide based strategies for applications of biomedical devices. Biosurf. Biotribol. 1, 177–201. doi: 10.1016/j.bsbt.2015.08.003

[ref192] Yong V. F. L. Soh M. M. Jaggi T. K. Mac Aogain M. Chotirmall S. H. (2018). The microbial endocrinology of *Pseudomonas aeruginosa*: inflammatory and immune perspectives. Arch. Immunol. Ther. Exp. 66, 329–339. doi: 10.1007/s00005-018-0510-1, PMID: 29541797

[ref193] Yu Z. Kong Y. Luo Z. Liu T. Lin J. (2019). Anti-bacterial activity of mutant chensinin-1 peptide against multidrug-resistant *Pseudomonas aeruginosa* and its effects on biofilm-associated gene expression. Exp. Ther. Med. 17, 2031–2038. doi: 10.3892/etm.2019.7182, PMID: 30867692 PMC6396000

[ref194] Yu S. Su T. Wu H. Liu S. Wang D. Zhao T.. (2015). PslG, a self-produced glycosyl hydrolase, triggers biofilm disassembly by disrupting exopolysaccharide matrix. Cell Res. 25, 1352–1367. doi: 10.1038/cr.2015.129, PMID: 26611635 PMC4670989

[ref195] Zhang J. He J. Zhai C. Ma L. Z. Gu L. Zhao K. (2018). Effects of PslG on the surface movement of *Pseudomonas aeruginosa*. Appl. Environ. Microbiol. 84, e00219-18. doi: 10.1128/AEM.00219-18, PMID: 29728385 PMC6007099

[ref196] Zhao X. L. Chen Z. G. Yang T. C. Jiang M. Wang J. Cheng Z. X.. (2021). Glutamine promotes antibiotic uptake to kill multidrug-resistant uropathogenic bacteria. Sci. Transl. Med. 13, eabj0716. doi: 10.1126/scitranslmed.abj0716, PMID: 34936385

[ref197] Zhao T. Zhang Y. Wu H. Wang D. Chen Y. Zhu M. J.. (2018). Extracellular aminopeptidase modulates biofilm development of *Pseudomonas aeruginosa* by affecting matrix exopolysaccharide and bacterial cell death. Environ. Microbiol. Rep. 10, 583–593. doi: 10.1111/1758-2229.12682, PMID: 30047246

[ref198] Zhu Y. McHale G. Dawson J. Armstrong S. Wells G. Han R.. (2022). Slippery liquid-Like solid surfaces with promising antibiofilm performance under both static and flow conditions. ACS Appl. Mater. Interfaces 14, 6307–6319. doi: 10.1021/acsami.1c14533, PMID: 35099179 PMC9096797

[ref199] Zhu X. Rice S. A. Barraud N. (2019). Nitric oxide and iron signaling cues have opposing effects on biofilm development in *Pseudomonas aeruginosa*. Appl. Environ. Microbiol. 85, e02175-18. doi: 10.1128/AEM.02175-18, PMID: 30478229 PMC6344620

